# Supplementary material for: Organisation and characteristics of out-of-hours primary care during a COVID-19 outbreak: A real-time observational study
Source: PLoS One. 2020 Aug 13;15(8):e0237629. doi: 10.1371/journal.pone.0237629 (PMC7425859; doi:10.1371/journal.pone.0237629)
Supplement: S1 File — (PDF) [file pone.0237629.s002.pdf]

Beschrijvende statistiek

kijk eerst aantallen na!

Tabulate

	Year		
ConsultType	2019	2020	All
huisbezoek	1132	1420	2552
raadpleging	6439	5311	11750
telefonisch consult	0	8924	8924
All	7571	15655	23226

751 rows have been excluded.

Tabulate

	Year			
	2019	2020	All	
SuspectedCovid 2	N	N	N	Column %
0	5687	8963	14650	63,09%
1	1879	6692	8571	36,91%
All	7566	15655	23221	100,00%

751 rows have been excluded.

Tabulate

	Year	
WeekendNr	2019	2020
1	1392	3564
2	1336	3630
3	1424	2903
4	1321	3610
5	2098	1948

751 rows have been excluded.

Waren alle zenders al goed aangesloten???

Tabulate

		ConsultType		
Sender	Year	huisbezoek	raadpleging	telefonisch consult
ACE	2020	81	320	1077
AOO	2019	102	945	0
	2020	63	243	949
AZU	2020	22	276	950
HDB	2019	236	1446	0
	2020	192	640	1014
LEU	2020	184	687	581
LRP	2019	72	368	0
	2020	107	283	887
TIE	2020	121	445	302
WSL	2019	306	1332	0
	2020	335	1084	1303
ZKE	2019	416	2348	0
	2020	315	1333	1861

751 rows have been excluded.

Leeftijd

Oneway Analysis of Leeftijd By Year

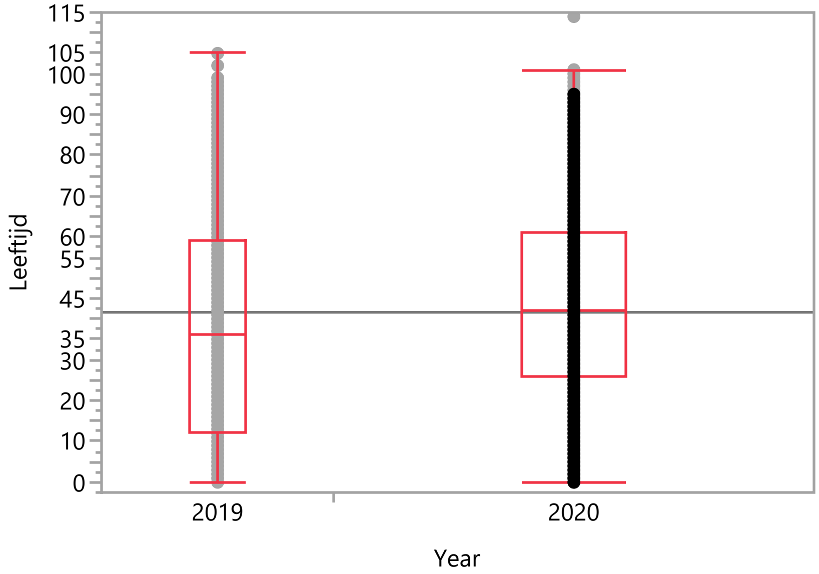

Excluded Rows 751

Geslacht

Tabulate

	Year					
	2019					
	ConsultType					
	huisbezoek		raadpleging		All	
Geslacht	Column %	N	Column %	N	Column %	N
F	53,53%	606	54,29%	3496	54,18%	4102
M	46,47%	526	45,71%	2943	45,82%	3469
All	100,00%	1132	100,00%	6439	100,00%	7571

751 rows have been excluded.

Leeftijd

Local Data Filter

15655 matching rows

☐ Inverse

Year (2)

2019 2020

Distributions

Leeftijd

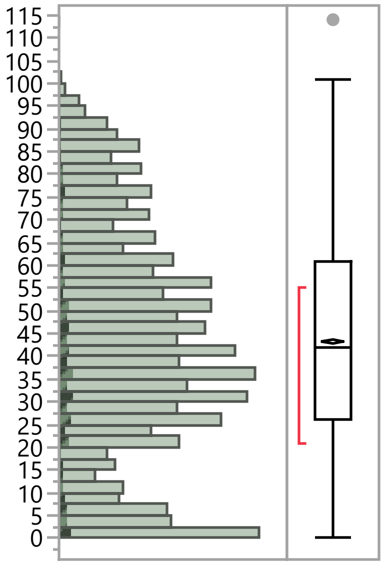

2020							
ConsultType							
huisbezoek		raadpleging		telefonisch consult		All	
Column %	N	Column %	N	Column %	N	Column %	N
60,28%	856	55,00%	2921	54,84%	4894	55,39%	8671
39,72%	564	45,00%	2390	45,16%	4030	44,61%	6984
100,00%	1420	100,00%	5311	100,00%	8924	100,00%	15655

Distributions

Leeftijd

Quantiles

100.0%	maximum	114
99.5%		96
97.5%		90
90.0%		79
75.0%	quartile	61
50.0%	median	42
25.0%	quartile	26
10.0%		6
2.5%		1
0.5%		1
0.0%	minimum	0

Summary Statistics

Mean	43,214692
Std Dev	24,838625
Std Err Mean	0,1985185
Upper 95% Mean	43,603811
Lower 95% Mean	42,825573
N	15655

Local Data Filter

8322 matching rows

Distributions

Local Data Filter

8322 matching rows

☐ Inverse

Year (2)

2019 2020

Distributions

Leeftijd

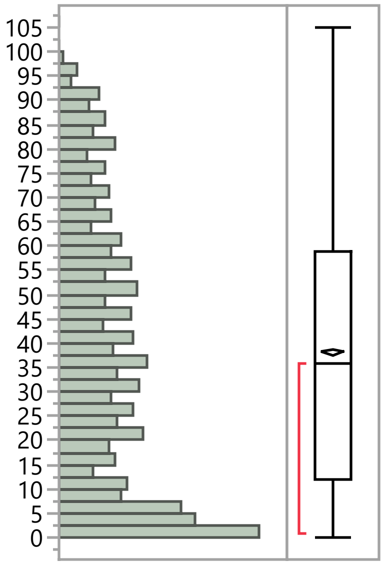

Quantiles

100.0%	maximum	105
99.5%		96
97.5%		90
90.0%		80
75.0%	quartile	59
50.0%	median	36
25.0%	quartile	12
10.0%		3
2.5%		1
0.5%		1
0.0%	minimum	0

Summary Statistics

Mean	38,271298
Std Dev	27,59717
Std Err Mean	0,317167
Upper 95% Mean	38,893034
Lower 95% Mean	37,649563
N	7571

Where(:ConsultType == "telefonisch consult")

Local Data Filter

8924 matching rows

☐ Inverse

Year (2)

2019 | 2020

Distributions

Leeftijd

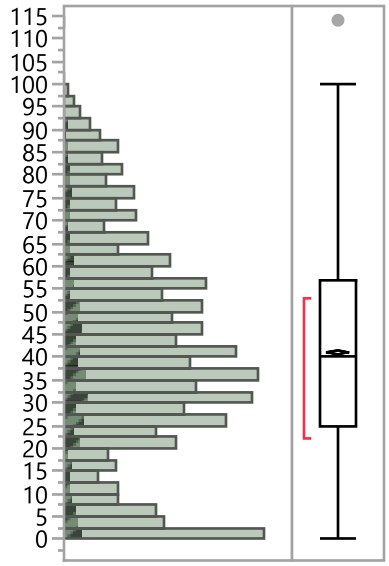

Quantiles

100.0%	maximum	114
99.5%		94
97.5%		88
90.0%		75
75.0%	quartile	57
50.0%	median	40
25.0%	quartile	25
10.0%		6
2.5%		1
0.5%		1
0.0%	minimum	0

Summary Statistics

Mean	41,0381
Std Dev	23,509494
Std Err Mean	0,2488648
Upper 95% Mean	41,525932
Lower 95% Mean	40,550267
N	8924

Where(:ConsultType == "raadpleging")

Local Data Filter

5311 matching rows

☐ Inverse

Year (2)

2019 2020

Distributions

Leeftijd

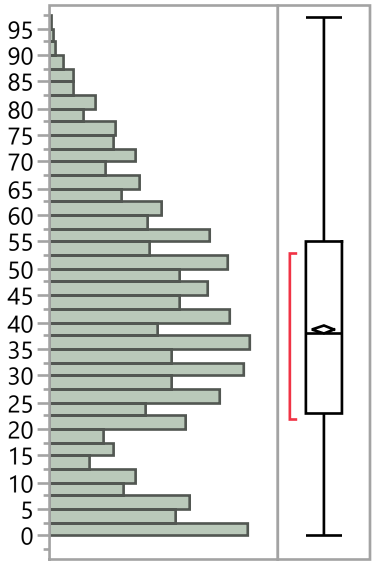

Quantiles

100.0%	maximum	97
99.5%		88
97.5%		81
90.0%		69
75.0%	quartile	55
50.0%	median	38
25.0%	quartile	23
10.0%		5
2.5%		1
0.5%		1
0.0%	minimum	0

Summary Statistics

Mean	38,705517
Std Dev	22,246409
Std Err Mean	0,3052613
Upper 95% Mean	39,303954
Lower 95% Mean	38,107079
N	5311

Where(:ConsultType == "huisbezoek")

Local Data Filter

1420 matching rows

☐ Inverse

Year (2)

2019 2020

Distributions

Leeftijd

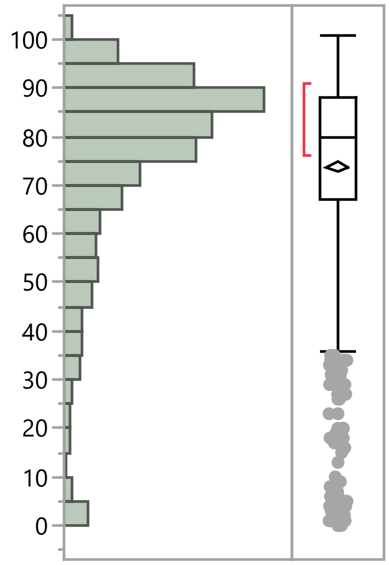

Quantiles

100.0%	maximum	101
99.5%		100
97.5%		97
90.0%		92
75.0%	quartile	88
50.0%	median	80
25.0%	quartile	67
10.0%		44,1
2.5%		5,525
0.5%		1
0.0%	minimum	0

Summary Statistics

Mean	73,758451
Std Dev	20,956584
Std Err Mean	0,55613
Upper 95% Mean	74,849376
Lower 95% Mean	72,667525
N	1420

aantal pt per wachtpost per jaar

Tabulate

	Year	
Sender	2019	2020
ACE	0	1478
AOO	1047	1255
AZU	0	1248
HDB	1682	1846
LEU	0	1452
LRP	440	1277
TIE	0	868
WSL	1638	2722
ZKE	2764	3509

751 rows have been excluded.

aantal pt per weekend

Tabulate

		ConsultType			
Year	WeekendNr	huisbezoek	raadpleging	telefonisch consult	All
2019	1	224	1168	0	1392
	2	187	1149	0	1336
	3	205	1219	0	1424
	4	200	1121	0	1321
	5	316	1782	0	2098
	All	1132	6439	0	7571
2020	1	274	1051	2239	3564
	2	309	1062	2259	3630
	3	292	994	1617	2903
	4	370	1424	1816	3610
	5	175	780	993	1948
	All	1420	5311	8924	15655

751 rows have been excluded.

hebben patienten eerst gebeld?, alleen relevant voor 2020

Local Data Filter

15655 matching rows

☐ Inverse

Year (2)

2019 2020

Tabulate

Year = 2020

	Sende														
	ACE			AOO			AZU			HDB			LEU		
	PriorCall			PriorCall			PriorCall			PriorCall			PriorCall		
	0	1	All	0	1	All	0	1	All	0	1	All	0	1	All
	125	276	401	99	207	306	106	192	298	520	312	832	673	198	871

8322 rows have been excluded.

Of ze eerst bellen hangt af van lokale organiatie. het laagste in Antwerpen Centrum waar ook gewone zorg wordt teruggebeld.

r

LRP			TIE			WSL			ZKE			All
PriorCall			PriorCall			PriorCall			PriorCall			
0	1	All	0	1	All	0	1	All	0	1	All	
135	255	390	446	120	566	922	497	1419	1056	592	1648	6731

Local Data Filter

15655 matching rows

☐ Inverse

Year (2)

2019 2020

Tabulate

Year = 2020

	PriorCall		
	0	1	All
**Sender**	**Row %**	**Row %**	**Row %**
ACE	31,17%	68,83%	100,00%
AOO	32,35%	67,65%	100,00%
AZU	35,57%	64,43%	100,00%
HDB	62,50%	37,50%	100,00%
LEU	77,27%	22,73%	100,00%
LRP	34,62%	65,38%	100,00%
TIE	78,80%	21,20%	100,00%
WSL	64,98%	35,02%	100,00%
ZKE	64,08%	35,92%	100,00%
All	60,64%	39,36%	100,00%

8322 rows have been excluded.

Local Data Filter

5311 matching rows

☐ Inverse

Year (2)

2019 2020

ConsultType (3)

huisbezoek (2575)

raadpleging (12477)

telefonisch consult (8925)

Tabulate

(Year = 2020) and (ConsultType = raadpleging)

	PriorCall					
	0		1		All	
**SuspectedCovid 2**	**N**	**Row %**	**N**	**Row %**	**N**	**Row %**
0	2652	68,87%	1199	31,13%	3851	100,00%
1	433	29,66%	1027	70,34%	1460	100,00%
All	3085	58,09%	2226	41,91%	5311	100,00%

18666 rows have been excluded.

Suspected cases

Local Data Filter

15655 matching rows

☐ Inverse

Year (2)

2019 2020

Tabulate

Year = 2020

	SuspectedCovid 2					
	0		1		All	
**ConsultType**	**N**	**Row %**	**N**	**Row %**	**N**	**Row %**
huisbezoek	1082	76,20%	338	23,80%	1420	100,00%
raadpleging	3851	72,51%	1460	27,49%	5311	100,00%
telefonisch consult	4030	45,16%	4894	54,84%	8924	100,00%
All	8963	57,25%	6692	42,75%	15655	100,00%

8322 rows have been excluded.

confirmed cases

Local Data Filter

86 matching rows

☐ Inverse

Tabulate

DiagnCod = 10118837

Local Data Filter

DiagnCod (2105)
10000065 (3)
10000083 (3)
10000094 (1)
10000127 (1)
10000211 (1)
10000224 (1)
10000225 (1)
10000257 (1)
10000264 (1)
10000267 (1)
10000288 (1)
10000293 (2)
10000296 (4)
10000386 (1)
10000390 (1)
10000407 (2)
10000509 (1)
10000516 (1)
10000532 (1)
10000549 (4)
10000552 (4)
10000699 (1)
10000713 (1)
10000726 (1)
10000730 (2)
10000750 (1)
10000751 (1)
10000758 (1)
10000760 (1)
10000788 (4)
10000822 (1)
10000854 (2)
10000866 (1)
10000867 (1)
10000900 (7)
10000917 (1)
10000920 (2)
10000922 (1)
10000926 (1)
10000933 (1)
10000950 (1)
10000956 (2)
10000958 (1)
10000970 (1)
10000973 (2)
10000990 (2)
10000991 (7)

Tabulate

	ConsultType		
	huisbezoek	raadpleging	telefonisch consult
	26	15	45

23891 rows have been excluded.

Local Data Filter

10001004 (1)
10001010 (4)
10001022 (2)
10001091 (148)
10001093 (2)
10001098 (1)
10001105 (3)
10001107 (1)
10001141 (1)
10001150 (13)
10001199 (1)
10001201 (21)
10001210 (5)
10001489 (1)
10001564 (2)
10001706 (1)
10001773 (2)
10001966 (1)
10002019 (1)
10002023 (1)
10002024 (4)
10002089 (14)
10002097 (4)
10002164 (1)
10002177 (1)
10002180 (1)
10002249 (1)
10002287 (1)
10002432 (8)
10002433 (1)
10002434 (3)
10002437 (7)
10002438 (7)
10002440 (14)
10002441 (87)
10002444 (1)
10002447 (115)
10002449 (1)
10002457 (37)
10002489 (3)
10002492 (5)
10002493 (2)
10002510 (1)
10002526 (22)
10002529 (3)
10002530 (1)
10002633 (1)
10002666 (5)
10002686 (1)

Local Data Filter

10002900 (1)
10002939 (1)
10003102 (5)
10003130 (2)
10003133 (1)
10003140 (1)
10003177 (2)
10003185 (1)
10003322 (1)
10003349 (18)
10003410 (36)
10003430 (5)
10003441 (2)
10003475 (1)
10003529 (6)
10003544 (1)
10003578 (1)
10004010 (16)
10004011 (8)
10004012 (2)
10004013 (1)
10004307 (1)
10004524 (2)
10004542 (1)
10004543 (1)
10004544 (1)
10004659 (1)
10004666 (4)
10004688 (2)
10004709 (3)
10004722 (1)
10004763 (6)
10004806 (173)
10004819 (2)
10004837 (3)
10004858 (1)
10004865 (13)
10004883 (2)
10004884 (52)
10004886 (17)
10004888 (3)
10004890 (8)
10005112 (1)
10005163 (1)
10005237 (2)
10005449 (1)
10005457 (1)
10005556 (1)
10005766 (7)

Local Data Filter

10006115 (1)
10006236 (2)
10006238 (1)
10006246 (1)
10006251 (1)
10006261 (19)
10006267 (3)
10006269 (2)
10006277 (1)
10006289 (7)
10006370 (78)
10006381 (1)
10006418 (2)
10006419 (1)
10006425 (2)
10006431 (3)
10006599 (1)
10006830 (1)
10006835 (1)
10006837 (1)
10006839 (14)
10006843 (10)
10006844 (5)
10006962 (1)
10007354 (5)
10007358 (8)
10007488 (4)
10007609 (3)
10007632 (2)
10007639 (1)
10007649 (1)
10007650 (1)
10007684 (6)
10007689 (3)
10007993 (43)
10008122 (1)
10008184 (3)
10008186 (3)
10008198 (1)
10008200 (1)
10008220 (1)
10008239 (1)
10008257 (2)
10008471 (1)
10008540 (1)
10008656 (1)
10008661 (3)
10008664 (1)
10008666 (31)

Local Data Filter

10008770 (4)
10008852 (1)
10008873 (1)
10008893 (1)
10009043 (1)
10009087 (7)
10009090 (2)
10009179 (1)
10009293 (1)
10009335 (7)
10009363 (50)
10009367 (4)
10009374 (1)
10009376 (1)
10009392 (1)
10009403 (2)
10009559 (1)
10009583 (3)
10009695 (3)
10009696 (1)
10009837 (1)
10009838 (1)
10009907 (1)
10009934 (1)
10009936 (1)
10009983 (7)
10010166 (1)
10010174 (2)
10010196 (2)
10010198 (2)
10010202 (3)
10010213 (1)
10010215 (1)
10010255 (20)
10010276 (2)
10010379 (1)
10010539 (10)
10010542 (1)
10010545 (1)
10010603 (28)
10010654 (2)
10010664 (1)
10010666 (9)
10010674 (1)
10010774 (4)
10011059 (13)
10011070 (1)
10011071 (1)
10011072 (1)

Local Data Filter

10011093 (2)
10011144 (3)
10011145 (10)
10011161 (6)
10011221 (1)
10011333 (1)
10011376 (1)
10011379 (1)
10011761 (1)
10011887 (2)
10011892 (3)
10011906 (2)
10011912 (23)
10011914 (2)
10012030 (2)
10012242 (1)
10012253 (1)
10012649 (1)
10012668 (2)
10012741 (1)
10012742 (1)
10012774 (7)
10012952 (1)
10012955 (15)
10012961 (5)
10013031 (2)
10013099 (3)
10013106 (3)
10013120 (1)
10013139 (3)
10013145 (1)
10013173 (1)
10013174 (1)
10013175 (1)
10013186 (1)
10013195 (35)
10013264 (1)
10013304 (1)
10013313 (4)
10013321 (3)
10013423 (1)
10013440 (1)
10013466 (1)
10013480 (1)
10013487 (1)
10013504 (4)
10013524 (5)
10013565 (4)
10013586 (9)

Local Data Filter

10013626 (1)
10013699 (1)
10013806 (1)
10013895 (1)
10014031 (1)
10014045 (2)
10014631 (1)
10014666 (1)
10014682 (16)
10014683 (5)
10014684 (1)
10014692 (1)
10014696 (4)
10014706 (145)
10014718 (1)
10014721 (3)
10014730 (1)
10014733 (1)
10014767 (2)
10014778 (1)
10014896 (1)
10014977 (5)
10014990 (6)
10015146 (1)
10015222 (8)
10015232 (1)
10015255 (1)
10015258 (1)
10015274 (236)
10015282 (1)
10015287 (1)
10015323 (2)
10015352 (38)
10015369 (3)
10015422 (1)
10015608 (2)
10015609 (27)
10015638 (13)
10015640 (1)
10015642 (6)
10015646 (4)
10015669 (1)
10015674 (1)
10015709 (1)
10015720 (1)
10015788 (1)
10015797 (1)
10015799 (3)
10015819 (1)

Local Data Filter

10015855 (21)
10015863 (5)
10015871 (1)
10015875 (3)
10015881 (10)
10015884 (2)
10015888 (4)
10015889 (1)
10015894 (1)
10015913 (1)
10015944 (1)
10016059 (3)
10016136 (1)
10016182 (1)
10016278 (3)
10016298 (1)
10016902 (2)
10016910 (4)
10016930 (3)
10016951 (1)
10016969 (1)
10017216 (6)
10017256 (1)
10017374 (1)
10017386 (1)
10017391 (1)
10017393 (1)
10017501 (47)
10017548 (1)
10017569 (3)
10017588 (1)
10017594 (3)
10017619 (1)
10017788 (1)
10017789 (1)
10017846 (33)
10017895 (1)
10017937 (1)
10017939 (21)
10017969 (3)
10017977 (2)
10017978 (43)
10017981 (2)
10018004 (9)
10018233 (1)
10018256 (3)
10018322 (1)
10018385 (1)
10018539 (1)

Local Data Filter

10018546 (9)
10018548 (3)
10018553 (1)
10018585 (1)
10018589 (1)
10018601 (1)
10018648 (1)
10018650 (1)
10018651 (2)
10018685 (1)
10018970 (1)
10019016 (3)
10019043 (1)
10019090 (1)
10019207 (1)
10019215 (1)
10019286 (2)
10019298 (6)
10019413 (1)
10019414 (1)
10019440 (1)
10019501 (4)
10019516 (1)
10019526 (1)
10019610 (1)
10019616 (4)
10019746 (15)
10019747 (1)
10019874 (1)
10019905 (1)
10019936 (2)
10020793 (1)
10020802 (3)
10020805 (1)
10020845 (1)
10020967 (1)
10021109 (1)
10021257 (1)
10021380 (4)
10021390 (1)
10021408 (5)
10021444 (36)
10021525 (1)
10021529 (71)
10021538 (1)
10021602 (1)
10021614 (1)
10021697 (2)
10021757 (38)

Local Data Filter

10021762 (6)
10021763 (1)
10021768 (3)
10021781 (1)
10021807 (1)
10021829 (12)
10021838 (16)
10021840 (2)
10021861 (10)
10021863 (1)
10021867 (3)
10021868 (12)
10021871 (1)
10021873 (1)
10021881 (1)
10021883 (44)
10021885 (3)
10021897 (1)
10021912 (1)
10021936 (1)
10021938 (1)
10021952 (19)
10021970 (1)
10021975 (1)
10021981 (5)
10022004 (11)
10022005 (1)
10022034 (13)
10022042 (21)
10022088 (1)
10022143 (13)
10022144 (3)
10022145 (8)
10022150 (1)
10022153 (1)
10022198 (10)
10022199 (2)
10022235 (1)
10022275 (2)
10022281 (1)
10022298 (1)
10022330 (4)
10022414 (1)
10022459 (1)
10022469 (1)
10022478 (2)
10022507 (1)
10022735 (5)
10022736 (1)

Local Data Filter

10022749 (1)
10022783 (5)
10022788 (2)
10022951 (1)
10022954 (12)
10022970 (1)
10023006 (3)
10023013 (1)
10023027 (1)
10023030 (1)
10023093 (1)
10023136 (1)
10023265 (1)
10023297 (1)
10023301 (31)
10023304 (1)
10023307 (1)
10023325 (1)
10023488 (1)
10023515 (1)
10023590 (1)
10023617 (1)
10023647 (1)
10023755 (328)
10023757 (95)
10023882 (1)
10023892 (2)
10024004 (1)
10024005 (13)
10024020 (4)
10024041 (1)
10024215 (5)
10024246 (10)
10024252 (1)
10024380 (1)
10024609 (7)
10024693 (2)
10024833 (1)
10024920 (15)
10024921 (1)
10025035 (4)
10025204 (16)
10025270 (1)
10025274 (6)
10025323 (1)
10025333 (5)
10025435 (3)
10025457 (4)
10025743 (1)

Local Data Filter

10025768 (1)
10025858 (1)
10025930 (17)
10026005 (39)
10026283 (1)
10026418 (1)
10026509 (28)
10026526 (1)
10026527 (1)
10026531 (1)
10026533 (79)
10026534 (1)
10026542 (2)
10026556 (1)
10026561 (1)
10026563 (1)
10026583 (6)
10026587 (10)
10026592 (1)
10026599 (1)
10026600 (1)
10026605 (1)
10026613 (1)
10026614 (10)
10026623 (4)
10026634 (1)
10026641 (1)
10026658 (3)
10026689 (1)
10026703 (4)
10026732 (184)
10026785 (6)
10026795 (1)
10027079 (26)
10027088 (13)
10027125 (21)
10027158 (1)
10027159 (1)
10027201 (1)
10027203 (4)
10027204 (1)
10027207 (1)
10027220 (3)
10027235 (1)
10027238 (2)
10027253 (1)
10027334 (1)
10027568 (1)
10027591 (6)

Local Data Filter

10027612 (51)
10027617 (3)
10027837 (2)
10027881 (1)
10027899 (1)
10027908 (2)
10027911 (4)
10027945 (1)
10027977 (5)
10028003 (6)
10028068 (311)
10028072 (11)
10028172 (23)
10028175 (3)
10028192 (10)
10028197 (2)
10028408 (27)
10028443 (6)
10028525 (1)
10028528 (2)
10028590 (1)
10028599 (1)
10028627 (1)
10028634 (1)
10028782 (1)
10028810 (1)
10028861 (3)
10028864 (16)
10028875 (1)
10028920 (3)
10029077 (1)
10029188 (1)
10029194 (17)
10029370 (1)
10029888 (1)
10030002 (3)
10030006 (6)
10030039 (1)
10030068 (2)
10030104 (1)
10030120 (1)
10030131 (75)
10030319 (8)
10030347 (1)
10030458 (9)
10030474 (1)
10030489 (1)
10030574 (9)
10030602 (1)

Local Data Filter

10030725 (1)
10030728 (1)
10030734 (1)
10030743 (42)
10030819 (1)
10030840 (2)
10030881 (1)
10030901 (124)
10030909 (1)
10030913 (2)
10030915 (1)
10030916 (1)
10030958 (6)
10030959 (4)
10030966 (2)
10030968 (4)
10030999 (3)
10031019 (1)
10031123 (1)
10031146 (2)
10031275 (1)
10031371 (2)
10031493 (1)
10031499 (6)
10031500 (2)
10031537 (3)
10031563 (26)
10031568 (6)
10031569 (4)
10031578 (3)
10031580 (1)
10031712 (46)
10031814 (3)
10031860 (2)
10031875 (1)
10031884 (13)
10031941 (1)
10032025 (1)
10032027 (1)
10032201 (1)
10032220 (203)
10032234 (2)
10032253 (2)
10032254 (2)
10032258 (1)
10032273 (12)
10032278 (4)
10032286 (1)
10032307 (8)

Local Data Filter

10032312 (1)
10032397 (2)
10032561 (1)
10032743 (1)
10032829 (1)
10032880 (2)
10032899 (5)
10032976 (5)
10033328 (3)
10033330 (2)
10033338 (8)
10033387 (1)
10033413 (1)
10033454 (2)
10033709 (14)
10033715 (2)
10033900 (1)
10033932 (17)
10033944 (1)
10034042 (12)
10034051 (2)
10034056 (1)
10034125 (2)
10034128 (1)
10034152 (1)
10034165 (10)
10034179 (1)
10034192 (1)
10034202 (6)
10034229 (1)
10034234 (3)
10034350 (2)
10034404 (1)
10034407 (1)
10034528 (3)
10034608 (6)
10034634 (4)
10034752 (1)
10034812 (1)
10034820 (2)
10034950 (2)
10035170 (1)
10035182 (277)
10035183 (52)
10035197 (1)
10035218 (1)
10035222 (2)
10035231 (157)
10035233 (1)

Local Data Filter

10035236 (2)
10035254 (87)
10035266 (1)
10035302 (2)
10035304 (2)
10035325 (1)
10035562 (1)
10035668 (1)
10036065 (26)
10036103 (1)
10036180 (1)
10036255 (1)
10036474 (4)
10036494 (2)
10036504 (3)
10036535 (1)
10036642 (1)
10036748 (1)
10037107 (2)
10037243 (1)
10037321 (2)
10037418 (1)
10037419 (10)
10037440 (2)
10037524 (1)
10037531 (1)
10037551 (2)
10037699 (2)
10037729 (3)
10037751 (1)
10037804 (1)
10037907 (10)
10037908 (1)
10038312 (3)
10038396 (6)
10038409 (4)
10038466 (1)
10038484 (3)
10038873 (1)
10038970 (2)
10038979 (3)
10038998 (4)
10039003 (10)
10039012 (1)
10039325 (1)
10039364 (1)
10039365 (1)
10039371 (1)
10039427 (3)

Local Data Filter

10039434 (1)
10039502 (1)
10039535 (1)
10039537 (3)
10039580 (2)
10039586 (5)
10039589 (1)
10039597 (12)
10039643 (1)
10039787 (1)
10039789 (32)
10039815 (3)
10039825 (3)
10039842 (1)
10039904 (12)
10039912 (4)
10039937 (3)
10039951 (1)
10040024 (12)
10040038 (6)
10040099 (1)
10040168 (10)
10040223 (1)
10040263 (1)
10040265 (31)
10040331 (1)
10040341 (3)
10040493 (3)
10040535 (14)
10040544 (1)
10040549 (6)
10040817 (7)
10040818 (2)
10040872 (1)
10040912 (1)
10040934 (1)
10040976 (2)
10041070 (1)
10041071 (6)
10041129 (2)
10041132 (2)
10041154 (47)
10041157 (1)
10041200 (1)
10041354 (3)
10041355 (1)
10041468 (13)
10041476 (1)
10041514 (1)

Local Data Filter

10041533 (1)
10041557 (5)
10041728 (433)
10041738 (2)
10041788 (1)
10041810 (3)
10041817 (1)
10041859 (147)
10041873 (1)
10041875 (2)
10041876 (24)
10041878 (1)
10041887 (13)
10041902 (1)
10041914 (25)
10041927 (21)
10041989 (1)
10042001 (29)
10042034 (32)
10042036 (3)
10042043 (18)
10042046 (3)
10042054 (2)
10042065 (1)
10042092 (1)
10042098 (4)
10042577 (1)
10042579 (1)
10042584 (2)
10042727 (1)
10042853 (2)
10043017 (2)
10043040 (1)
10043043 (1)
10043225 (1)
10043296 (4)
10043305 (7)
10043400 (1)
10043606 (162)
10043883 (1)
10043892 (157)
10044129 (14)
10044172 (1)
10044403 (26)
10044408 (2)
10044412 (13)
10044470 (1)
10044508 (3)
10044563 (1)

Local Data Filter

10044595 (6)
10044596 (2)
10044624 (2)
10044665 (1)
10044685 (6)
10044690 (1)
10044931 (6)
10044958 (26)
10044969 (1)
10044970 (2)
10044995 (1)
10045141 (1)
10045168 (5)
10045286 (4)
10045342 (79)
10045391 (2)
10045421 (2)
10045423 (6)
10045432 (2)
10045482 (6)
10045529 (5)
10045541 (2)
10045622 (45)
10045627 (1)
10045653 (356)
10045654 (35)
10045655 (1)
10045657 (1)
10045659 (2)
10045703 (1)
10045711 (2)
10045723 (1)
10045746 (1)
10045787 (1)
10045801 (18)
10045815 (1)
10045827 (1)
10045829 (1)
10045837 (11)
10045877 (97)
10045903 (3)
10045942 (5)
10045956 (1)
10045959 (4)
10045964 (65)
10045966 (15)
10045976 (1)
10045981 (2)
10045999 (1)

Local Data Filter

10046244 (1)
10046310 (20)
10046323 (1)
10046419 (1)
10046445 (1)
10046494 (9)
10046504 (2)
10046595 (1)
10046612 (1)
10046712 (66)
10046735 (3)
10046752 (1)
10046897 (1)
10046989 (1)
10047038 (7)
10047129 (1)
10047461 (3)
10047473 (1)
10047566 (1)
10047719 (1)
10047733 (1)
10047737 (2)
10047749 (1)
10047750 (1)
10047758 (1)
10047766 (2)
10047779 (1)
10047802 (31)
10047804 (25)
10047912 (1)
10048003 (30)
10048031 (28)
10048053 (1)
10048054 (1)
10048169 (4)
10048195 (1)
10048210 (13)
10048260 (1)
10048325 (1)
10048360 (1)
10048378 (1)
10048428 (1)
10048440 (2)
10048487 (135)
10048500 (1)
10048508 (278)
10048510 (1)
10048512 (6)
10048526 (3)

Local Data Filter

10048632 (4)
10048796 (1)
10048903 (1)
10049029 (8)
10049031 (5)
10049076 (1)
10049087 (5)
10049121 (19)
10049129 (1)
10049272 (8)
10049278 (1)
10049281 (20)
10049284 (1)
10049305 (677)
10049445 (17)
10049510 (1)
10049565 (83)
10049774 (6)
10049775 (6)
10049777 (4)
10049802 (1)
10049818 (1)
10049824 (18)
10049906 (2)
10050031 (4)
10050052 (135)
10050301 (2)
10050484 (1)
10050563 (3)
10050565 (1)
10050596 (2)
10050705 (2)
10051006 (1)
10051009 (19)
10051033 (2)
10051036 (1)
10051074 (2)
10051079 (1)
10051128 (1)
10051605 (4)
10051619 (2)
10051676 (1)
10051682 (1)
10051684 (12)
10051722 (1)
10051776 (2)
10052172 (5)
10052176 (8)
10052207 (16)

Local Data Filter

10052256 (1)
10052352 (2)
10052456 (2)
10052492 (5)
10052549 (1)
10052608 (1)
10052668 (2)
10052772 (1)
10052797 (1)
10052860 (2)
10053401 (2)
10053549 (1)
10053612 (24)
10053622 (1)
10053654 (2)
10053715 (1)
10053763 (1)
10053771 (11)
10053836 (1)
10053879 (23)
10053920 (9)
10054548 (1)
10055010 (1)
10055227 (1)
10055397 (1)
10055410 (14)
10055550 (9)
10055553 (12)
10055588 (1)
10055685 (34)
10055694 (1)
10055704 (1)
10055710 (1)
10056008 (2)
10056009 (1)
10056036 (1)
10056050 (116)
10056053 (14)
10056133 (7)
10056246 (1)
10056270 (2)
10056286 (1)
10056317 (1)
10056366 (3)
10056396 (14)
10056806 (2)
10056875 (1)
10056973 (1)
10056975 (83)

Local Data Filter

10056986 (1)
10056999 (1)
10057050 (3)
10057110 (6)
10057261 (63)
10057264 (2)
10057496 (1)
10057503 (1)
10057569 (1)
10057974 (1)
10057993 (15)
10058031 (1)
10058365 (3)
10058419 (6)
10058459 (353)
10058520 (5)
10058526 (30)
10058540 (47)
10058545 (2)
10058550 (4)
10058566 (1)
10059020 (2)
10059021 (4)
10059117 (2)
10059125 (4)
10059456 (1)
10059500 (6)
10059540 (3)
10059584 (2)
10060075 (1)
10060132 (1)
10060133 (1)
10060196 (2)
10060310 (3)
10060311 (3)
10060368 (1)
10060375 (73)
10060653 (4)
10060657 (4)
10060658 (6)
10060703 (28)
10061361 (17)
10061372 (1)
10061394 (6)
10061436 (1)
10061511 (3)
10061534 (4)
10061558 (1)
10061559 (4)

Local Data Filter

10061567 (1)
10061568 (5)
10061636 (1)
10061690 (1)
10061817 (1)
10062081 (1)
10062136 (2)
10062160 (9)
10062226 (1)
10062229 (3)
10062846 (4)
10062946 (1)
10062947 (1)
10063145 (1)
10063177 (1)
10063184 (4)
10063197 (3)
10063218 (1)
10063230 (1)
10063233 (1)
10063288 (1)
10063878 (5)
10071091 (1)
10071101 (1)
10071124 (7)
10071125 (2)
10071163 (4)
10071336 (11)
10071351 (10)
10071374 (3)
10071381 (1)
10071390 (2)
10071635 (3)
10071668 (1)
10071674 (1)
10071770 (1)
10071913 (10)
10071940 (4)
10071977 (2)
10071986 (13)
10072167 (5)
10072177 (1)
10072258 (1)
10072309 (9)
10072412 (7)
10072555 (1)
10072579 (1)
10072599 (1)
10072601 (7)

Local Data Filter

10072607 (9)
10072741 (1)
10072743 (1)
10072812 (1)
10073207 (1)
10073317 (63)
10073417 (1)
10073459 (6)
10073463 (1)
10073558 (1)
10073597 (30)
10073615 (4)
10073693 (1)
10073697 (1)
10073718 (1)
10073742 (8)
10073743 (15)
10073947 (1)
10074011 (5)
10074087 (6)
10074157 (2)
10074337 (1)
10074468 (1)
10074473 (5)
10074474 (104)
10074485 (6)
10074546 (13)
10074705 (16)
10074709 (140)
10074786 (6)
10074791 (1)
10074794 (5)
10074796 (2)
10074805 (5)
10074807 (2)
10074832 (4)
10074836 (15)
10074844 (1)
10074939 (2)
10075135 (4)
10075168 (7)
10075170 (34)
10075194 (23)
10075349 (3)
10075363 (7)
10075400 (33)
10075422 (1)
10075445 (2)
10075468 (1)

Local Data Filter

10075476 (1)
10075479 (4)
10075493 (110)
10075495 (11)
10075504 (3)
10075506 (2)
10075565 (2)
10075569 (2)
10075592 (4)
10075830 (1)
10075932 (2)
10075983 (3)
10076081 (1)
10076197 (4)
10076240 (1)
10076543 (1)
10076666 (6)
10076716 (22)
10076737 (2)
10076740 (2)
10076768 (2)
10076794 (2)
10076809 (1)
10076810 (1)
10076814 (1)
10076853 (21)
10076854 (6)
10076860 (2)
10076883 (3)
10076984 (1)
10077040 (1)
10077069 (7)
10077183 (3)
10077238 (2)
10077303 (3)
10077318 (4)
10077373 (1)
10077390 (4)
10077458 (33)
10077490 (47)
10077508 (18)
10077645 (2)
10077685 (9)
10077686 (8)
10077690 (6)
10077691 (3)
10077719 (1)
10077747 (1)
10078195 (2)

Local Data Filter

10078262 (1)
10078497 (3)
10078530 (2)
10078564 (1)
10078572 (1)
10078587 (2)
10078590 (20)
10078607 (1)
10078631 (6)
10078659 (1)
10078839 (1)
10078900 (2)
10078925 (4)
10079008 (1)
10079068 (1)
10079070 (17)
10079075 (3)
10079129 (5)
10079162 (2)
10079324 (4)
10079396 (1)
10079450 (3)
10079544 (4)
10079778 (1)
10079799 (1)
10079930 (3)
10079986 (1)
10080047 (1)
10080048 (1)
10080173 (1)
10080184 (2)
10080305 (1)
10080326 (3)
10080406 (4)
10080436 (1)
10080445 (142)
10080459 (6)
10080462 (19)
10080465 (3)
10080468 (5)
10080485 (79)
10080486 (212)
10080511 (1)
10080514 (7)
10080527 (14)
10080552 (3)
10080553 (12)
10080554 (6)
10080556 (1)

Local Data Filter

10080557 (1)
10080558 (1)
10080582 (1)
10080583 (107)
10080585 (7)
10080592 (1)
10080641 (46)
10080659 (56)
10080660 (1)
10080673 (7)
10080675 (2)
10080680 (135)
10080682 (7)
10080685 (2)
10080686 (5)
10080689 (9)
10080694 (103)
10080699 (97)
10080705 (7)
10080706 (104)
10080710 (4)
10080722 (19)
10080725 (37)
10080729 (1)
10080754 (1)
10080793 (1)
10080810 (1)
10080857 (1)
10080865 (2)
10080869 (1)
10081055 (4)
10081247 (1)
10081274 (2)
10081285 (2)
10081331 (1)
10081456 (1)
10081458 (2)
10081596 (155)
10081619 (4)
10081830 (1)
10081834 (5)
10081846 (1)
10081953 (7)
10082065 (4)
10082068 (1)
10082105 (18)
10082147 (1)
10082190 (1)
10082541 (5)

Local Data Filter

10082719 (1)
10082775 (1)
10082798 (12)
10083069 (1)
10083124 (1)
10083131 (1)
10083136 (4)
10083151 (15)
10083470 (2)
10083516 (1)
10083544 (1)
10083545 (6)
10083585 (4)
10083685 (1)
10083733 (1)
10083943 (1)
10083944 (17)
10083946 (6)
10084099 (2)
10084158 (2)
10084253 (2)
10084266 (1)
10084320 (3)
10084322 (4)
10084327 (12)
10084334 (1)
10084375 (1)
10084484 (9)
10084631 (1)
10084839 (3)
10084907 (1)
10085041 (1)
10085062 (1)
10085151 (1)
10085155 (1)
10085197 (1)
10085198 (1)
10085208 (3)
10085220 (1)
10085231 (1)
10085259 (52)
10085260 (4)
10085411 (1)
10085496 (8)
10085621 (1)
10085737 (3)
10085768 (1)
10085806 (1)
10085830 (29)

Local Data Filter

10085841 (2)
10085915 (1)
10086081 (1)
10086084 (9)
10086091 (1)
10086238 (3)
10086344 (1)
10086404 (1)
10086460 (1)
10086518 (1)
10086533 (13)
10086860 (2)
10087076 (2)
10087078 (63)
10087203 (6)
10087206 (1)
10087267 (41)
10087268 (9)
10087278 (16)
10087298 (25)
10087310 (1)
10087335 (1)
10087342 (6)
10087459 (7)
10087469 (1)
10087470 (1)
10087508 (1)
10087574 (2)
10087595 (2)
10087626 (1)
10087712 (16)
10087801 (1)
10087824 (1)
10087909 (1)
10087957 (1)
10088050 (2)
10088337 (6)
10088339 (1)
10088364 (2)
10088373 (5)
10088506 (1)
10088523 (22)
10088551 (1)
10088558 (3)
10088579 (1)
10088599 (1)
10088889 (1)
10088929 (5)
10088955 (7)

Local Data Filter

10088966 (69)
10089096 (2)
10089265 (1)
10089275 (1)
10089483 (1)
10089566 (1)
10089874 (5)
10090338 (1)
10090345 (2)
10090349 (1)
10090382 (1)
10090449 (1)
10090586 (1)
10090600 (1)
10090602 (2)
10090651 (3)
10090720 (121)
10090725 (1)
10090868 (1)
10090876 (8)
10090951 (2)
10090987 (3)
10090990 (1)
10091032 (2)
10091055 (1)
10091058 (3)
10091126 (10)
10091166 (2)
10091167 (1)
10091194 (1)
10091204 (395)
10091257 (1)
10091325 (1)
10091351 (1)
10091362 (2)
10091384 (5)
10091425 (1)
10091499 (1)
10091547 (1)
10091549 (2)
10091572 (6)
10091658 (1)
10091668 (2)
10091672 (2)
10091678 (6)
10091689 (1)
10091949 (1)
10092164 (1)
10092180 (7)

Local Data Filter

10092181 (8)
10092225 (2)
10092268 (3)
10092269 (2)
10092270 (2)
10092346 (5)
10092372 (1)
10092404 (38)
10092408 (4)
10092411 (2)
10092664 (1)
10092827 (1)
10093178 (19)
10093182 (2)
10093436 (2)
10093632 (11)
10093795 (1)
10093824 (7)
10094128 (1)
10094164 (1)
10094319 (1)
10094530 (1)
10094556 (2)
10094558 (14)
10094770 (2)
10094799 (1)
10095758 (35)
10096128 (4)
10096158 (6)
10096161 (1)
10096171 (1)
10096204 (3)
10096210 (1)
10096212 (436)
10096262 (2)
10096474 (27)
10096490 (3)
10096510 (2)
10096517 (2)
10096570 (2)
10096572 (1)
10096860 (23)
10096893 (3)
10096925 (1)
10096934 (1)
10096937 (1)
10096978 (13)
10096984 (1)
10096985 (3)

Local Data Filter

10096989 (6)
10096997 (1)
10097007 (2)
10097011 (1)
10097016 (1)
10097059 (4)
10097188 (1)
10097202 (2)
10097268 (1)
10097294 (3)
10097350 (1)
10097489 (1)
10097530 (1)
10097643 (1)
10097703 (26)
10097729 (1)
10097808 (3)
10097839 (5)
10097842 (4)
10098095 (2)
10098097 (9)
10098100 (1)
10098195 (1)
10098203 (4)
10098312 (1)
10098359 (306)
10098366 (1)
10098373 (2)
10098421 (29)
10098428 (1)
10098783 (25)
10098800 (1)
10098815 (3)
10098827 (1)
10099041 (70)
10099113 (2)
10099116 (1)
10099202 (3)
10099203 (1)
10099284 (1)
10099395 (4)
10099399 (1)
10099549 (4)
10099596 (1)
10099600 (13)
10099619 (2)
10099960 (1)
10101321 (4)
10101367 (37)

Local Data Filter

10101407 (26)
10101408 (64)
10101412 (1)
10101414 (2)
10101420 (1)
10101421 (3)
10101548 (1)
10101693 (1)
10101697 (1)
10101785 (1)
10101856 (2)
10102033 (1)
10102048 (3)
10102175 (5)
10102266 (1)
10102267 (1)
10102272 (1)
10102279 (2)
10102412 (6)
10102421 (4)
10102425 (107)
10102910 (1)
10102961 (1)
10102970 (6)
10103007 (2)
10103081 (9)
10103160 (1)
10103248 (71)
10103304 (2)
10103321 (1)
10103337 (1)
10103518 (1)
10103784 (3)
10103992 (1)
10104043 (10)
10104049 (1)
10104070 (1)
10104087 (2)
10104117 (8)
10104410 (75)
10104413 (21)
10104419 (51)
10104528 (2)
10104540 (11)
10104555 (1)
10104594 (1)
10104623 (31)
10104797 (3)
10104810 (1)

Local Data Filter

10104913 (1)
10104919 (1)
10104954 (63)
10104964 (7)
10105077 (30)
10105111 (9)
10105114 (1)
10105328 (8)
10105426 (1)
10105430 (2)
10105543 (1)
10105584 (58)
10105593 (4)
10105600 (9)
10105613 (12)
10105614 (27)
10105707 (2)
10105714 (5)
10105717 (1)
10105973 (2)
10105975 (5)
10106001 (1)
10106055 (1)
10106096 (4)
10106122 (7)
10106153 (2)
10106181 (3)
10106207 (1)
10106220 (1)
10106485 (1)
10106497 (1)
10106641 (7)
10106864 (2)
10106900 (2)
10106934 (1)
10107089 (3)
10107132 (6)
10107243 (1)
10107421 (6)
10107442 (7)
10107444 (2)
10107506 (1)
10107563 (3)
10107609 (7)
10107622 (2)
10107646 (2)
10107650 (1)
10107653 (1)
10107662 (1)

Local Data Filter

10107664 (1)
10107666 (2)
10107672 (1)
10107674 (8)
10107694 (2)
10107730 (329)
10107774 (2)
10107791 (2)
10107793 (2)
10107794 (2)
10107811 (14)
10108033 (1)
10108037 (4)
10108038 (1)
10108056 (1)
10108361 (1)
10108679 (1)
10108757 (4)
10108801 (1)
10108910 (2)
10108913 (1)
10108925 (7)
10108956 (1)
10109119 (1)
10109130 (4)
10109135 (1)
10109398 (6)
10109418 (3)
10110253 (1)
10110296 (1)
10110319 (1)
10110331 (1)
10110339 (1)
10110508 (3)
10110637 (3)
10110875 (1)
10110965 (2)
10110986 (1)
10111013 (3)
10111017 (2)
10111021 (1)
10111023 (1)
10111049 (1)
10111071 (2)
10111089 (2)
10111113 (7)
10111114 (2)
10111116 (13)
10111125 (6)

Local Data Filter

10111181 (7)
10111286 (1)
10111305 (2)
10111329 (3)
10111332 (2)
10111369 (2)
10111380 (1)
10111415 (71)
10111438 (1)
10111444 (2)
10111460 (3)
10111537 (1)
10111538 (3)
10111539 (1)
10111546 (1)
10111575 (1)
10111695 (1)
10111698 (1)
10111746 (2)
10111787 (1)
10111825 (1)
10111918 (1)
10111953 (1)
10111956 (1)
10111962 (1)
10111972 (2)
10112164 (6)
10112518 (1)
10112532 (1)
10113037 (1)
10113407 (1)
10113600 (3)
10113681 (1)
10114058 (1)
10114199 (1)
10114200 (1)
10114220 (1)
10114236 (1)
10114322 (4)
10114357 (2)
10114359 (1)
10114363 (1)
10114400 (1)
10114469 (5)
10114471 (2)
10114538 (1)
10114610 (1)
10114641 (27)
10114880 (1)

Local Data Filter

10114898 (2)
10115143 (1)
10115145 (1)
10115155 (1)
10115157 (1)
10115166 (1)
10115216 (1)
10115217 (3)
10115221 (5)
10115246 (1)
10115261 (1)
10115541 (1)
10115572 (1)
10115608 (1)
10115629 (1)
10115955 (2)
10115959 (1)
10116023 (7)
10116040 (219)
10116049 (1)
10116053 (1)
10116054 (2)
10116143 (1)
10116216 (1)
10116350 (3)
10116434 (1)
10116739 (1)
10116953 (1)
10117062 (1)
10117064 (1)
10117261 (1)
10117274 (1)
10117276 (1)
10117291 (16)
10117295 (1)
10117301 (1)
10117364 (1)
10117415 (2)
10117450 (23)
10117451 (9)
10117509 (1)
10117640 (2)
10117866 (1)
10117960 (3)
10118020 (1)
10118022 (1)
10118023 (3)
10118034 (3)
10118035 (1)

Local Data Filter

10118042 (2)
10118107 (1)
10118490 (1)
10118678 (2)
10118684 (215)
10118687 (5)
10118689 (1)
10118760 (1)
10118792 (3)
10118827 (1)
10118835 (14)
10118837 (86)
10118856 (3131)
10118857 (29)
10118858 (254)
10118860 (4)
10118873 (1)
10118887 (2)
10118916 (206)
10118993 (1)
10119142 (1)
10119214 (26)
10119716 (1)
10119717 (3)
10119725 (1)
10120032 (1)
10120407 (1)
10120688 (1)
10120758 (1)
10120835 (1)
10120954 (1)
10120980 (1)
10121154 (4)
10121191 (1)
10121273 (6)
10121278 (1)
10121282 (1)
10121317 (2)
10121319 (2)
10121339 (3)
10121375 (2)
10121646 (1)
10121977 (13)
10121979 (1)
10121980 (1)
10121981 (14)
10122013 (21)
10122027 (9)
10122091 (1)

Local Data Filter

10122200 (1)
10122249 (1)
10122700 (1)
10122702 (1)
10122703 (1)
10122712 (1)
10122908 (1)
10122910 (1)
10122930 (5)
10123030 (10)
10123033 (1)
10123188 (2)
10123312 (5)
10123398 (1)
10123680 (1)
10124190 (2)
10124286 (2)
10124834 (1)
10124839 (2)
10124857 (2)
10125095 (1)
10125172 (3)
10125219 (2)
10125289 (2)
10125290 (6)
10125293 (4)
15000085 (1)
15000095 (1)
15000097 (1)
15000101 (1)
15000218 (1)
15000285 (1)
15000368 (1)
15000479 (1)
15000843 (5)
15000918 (1)
15000966 (1)
15001167 (1)
15001338 (1)
15001339 (1)
15001349 (1)
15001351 (5)
15001377 (1)
15001389 (1)
15001463 (1)
15001505 (7)
15001537 (5)
15001540 (2)
15001550 (12)

Local Data Filter

15001584 (1)
15001585 (8)
15001597 (1)
15001607 (1)
15001666 (1)
15001797 (1)
15001892 (144)
15001902 (1)
15001920 (1)
15001927 (1)
15001928 (3)
15001932 (1)
15001991 (1)
15001994 (2)
15002070 (2)
15002115 (1)
15002159 (3)
15002161 (1)
15002176 (1)
15002284 (1)
15002285 (1)
15002371 (1)
15002418 (1)
15002605 (1)
15002671 (9)
15002789 (3)
15002793 (1)
15002797 (2)
15002880 (1)
15002977 (1)
15003020 (1)
15003082 (4)
15003192 (1)
15003204 (1)
15003257 (1)
15003514 (3)
15003689 (4)
15003700 (50)
15003734 (1)
15003748 (8)
15003777 (20)
15003861 (21)
15003868 (3)
15003869 (1)
15003872 (1)
15003911 (4)
15003983 (4)
15004005 (2)
15004016 (1)

Local Data Filter

15004026 (1)
15004032 (1)
15004034 (42)
15004078 (6)
15004089 (11)
15004093 (154)
15004098 (2)
15004142 (2)
15004173 (2)
15004203 (1)
15004210 (1)
15004229 (1)
15004233 (1)
15004241 (1)
15004282 (1)
15004284 (1)
15004287 (1)
15004306 (4)
15004320 (2)
15004321 (1)
15004328 (1)
15004347 (2)
15004348 (1)
15004384 (147)
15004394 (2)
15004800 (3)
15004801 (4)
15004803 (1)
15004804 (100)
15004834 (5)
15004837 (1)
15004876 (2)
15004882 (2)
15004884 (14)
15004910 (1)
15004911 (2)
15004915 (2)
15004933 (7)
15004934 (2)
15004937 (4)
15004939 (1)
15004954 (7)
15004955 (2)
15005001 (1)
15005023 (4)
15005036 (1)
15005045 (2)
15005058 (1)
15005071 (1)

Local Data Filter

15005086 (1)
15005088 (2)
15005114 (2)
15005115 (1)
15005116 (1)
15005119 (1)
15005242 (1)
15005246 (45)
15005273 (2)
15005277 (2)
15005279 (1)
15005282 (4)
15005283 (1)
15005315 (1)
15005317 (1)
15005321 (7)
15005322 (41)
15005323 (5)
15005325 (2)
15005326 (4)
15005327 (22)
15005329 (1)
15005341 (2)
15005345 (8)
15005360 (1)
20000001 (6)
20000002 (3)
20000012 (2)
20000019 (1)
20000036 (1)
20000039 (1)
20000076 (28)
20000235 (1)
20000255 (15)
30000001 (2)
30000029 (1)
30000051 (1)
30000224 (1)
30000403 (1)
30000479 (1)
30000493 (1)
30000593 (1)
30000829 (1)
30001068 (1)
30001153 (5)
30001231 (1)
30001636 (1)
30001711 (1)
40001128 (66)

Local Data Filter

Meest frequente diagnoses

Local Data Filter

15655 matching rows

☐ Inverse

Year (2)

2019 2020

Tabulate

Year = 2020

DiagnIBUIFinal	N
10118856	3131
10049305	599
10041728	365
10096212	317
10028068	274
10091204	257
10118858	254
10048508	223
10107730	211
10023755	190
10080486	162
10118916	160
10116040	156
10035182	136
10045653	133
10043892	128
10026732	125
10043606	119
10035231	113
10080445	111
10004806	110
15001892	106
15004384	106
10080680	105
10058459	104
10098359	102
10041859	101
10118684	100
10014706	99
10074474	95
10015274	91
10080706	88
10118837	86
10001091	84
10075493	80
10049565	76
10081596	75
10080699	73
10006370	71
10056975	71
10080694	69
10030901	68
[illegible]	[illegible]

Tabulate

DiagnIBUIFinal	N
15004804	66
10056050	65
10045877	63
10074709	61
10080583	61
10045342	60
10102425	60
10050052	58
10057261	56
10032220	53
10080659	52
10080485	51
10002447	48
40001128	48
10058540	47
10021529	46
10105584	46
10104410	45
15004093	45
10088966	42
10099041	42
10104954	42
10027612	41
10077490	41
10048487	40
10060375	40
10041154	39
10046712	39
10087078	39
10090720	39
10026533	38
10101408	38
10101367	37
10111415	37
10003410	36
10009363	36
10023757	36
10104419	35
15005322	35
10073317	33
10085259	33
15003700	33
10007993	29
10017501	29
10030131	29
10030743	29
10118857	29
10092404	28
[illegible]	[illegible]

Tabulate

DiagnIBUIFinal	N
10004884	27
10080641	27
15004034	27
10021883	26
10058526	26
10013195	25
10026005	25
10104623	25
10039789	24
10040265	24
10055685	24
10073597	24
10085830	24
10075170	23
10035254	22
10048003	22
10075400	22
10105077	22
10114641	22
10023301	21
10031712	21
10036065	21
10060703	21
10077458	21
10041914	20
10047802	20
10096474	20
10021757	19
10027125	19
10080725	19
10002441	18
10010603	18
10017978	18
10028172	18
10045654	18
10049281	18
10087267	18
10095758	18
10035183	17
10045622	17
10008666	16
10017846	16
10041876	16
10042001	16
10096860	16
10097703	16
15003861	16
10003349	15
[illegible]	[illegible]

Tabulate

DiagnIBUIFinal	N
10041927	15
10042034	15
10044403	15
10048031	15
10075194	15
10015609	14
10033932	14
10042043	14
10101407	14
10119214	14
10006261	13
10021952	13
10025204	13
10028408	13
10061361	13
10080462	13
10080722	13
10082105	13
10121981	13
10006839	12
10044958	12
10078590	12
10079070	12
10094558	12
10103248	12
10122013	12
10002089	11
10019746	11
10022042	11
10026509	11
10027088	11
10031884	11
10047804	11
10049445	11
10053612	11
10053879	11
10083944	11
10088523	11
10104413	11
10117291	11
15003777	11
10004886	10
10010255	10
10015855	10
10024920	10
10044129	10
10073743	10
10123030	10
[illegible]	[illegible]

Tabulate

DiagnIBUIFinal	N
10001150	9
10001201	9
10002457	9
10021444	9
10027079	9
10028072	9
10029194	9
10031563	9
10033709	9
10034042	9
10034165	9
10037907	9
10039904	9
10040024	9
10040535	9
10041887	9
10049121	9
10051684	9
10053771	9
10056053	9
10071336	9
10072607	9
10074546	9
10077508	9
10080553	9
10093632	9
10098783	9
10105614	9
15004089	9
15005327	9
10004865	8
10006843	8
10007358	8
10013586	8
10014682	8
10028864	8
10041468	8
10044412	8
10046310	8
10048210	8
10052207	8
10056396	8
10057993	8
10071986	8
10074705	8
10083151	8
10087298	8
10087712	8
[illegible]	[illegible]

Tabulate

DiagnIBUIFinal	N
10091126	8
10098421	8
10105328	8
15004884	8
10004010	7
10010666	7
10011912	7
10018546	7
10021829	7
10021838	7
10022143	7
10022954	7
10024005	7
10024609	7
10025930	7
10039597	7
10045964	7
10062160	7
10075495	7
10077685	7
10080527	7
10084484	7
10086533	7
10090876	7
10093178	7
10099600	7
10106641	7
10107811	7
10111113	7
15001550	7
15001585	7
15002671	7
15003748	7
15005246	7
10009087	6
10009335	6
10011059	6
10012955	6
10015352	6
10015881	6
10017939	6
10021868	6
10022004	6
10022034	6
10026587	6
10030458	6
10030574	6
10037419	6
[illegible]	[illegible]

Tabulate

DiagnIBUIFinal	N
10039003	6
10044685	6
10049775	6
10055410	6
10060658	6
10071351	6
10072309	6
10080459	6
10080514	6
10080585	6
10080673	6
10080689	6
10080705	6
10082798	6
10084327	6
10086084	6
10087203	6
10088955	6
10091572	6
10096978	6
10103081	6
10104540	6
10107674	6
10121977	6
10122027	6
10125290	6
15001505	6
15004954	6
15005321	6
10002438	5
10002526	5
10003102	5
10009983	5
10010539	5
10011145	5
10012774	5
10014990	5
10015638	5
10017216	5
10022198	5
10024246	5
10030006	5
10034608	5
10045423	5
10049029	5
10055553	5
10057110	5
10058419	5
[illegible]	[illegible]

Tabulate

DiagnIBUIFinal	N
10058520	5
10061394	5
10071124	5
10071913	5
10074087	5
10076716	5
10077686	5
10078631	5
10088337	5
10091678	5
10097839	5
10098097	5
10102970	5
10104043	5
10105111	5
10109398	5
10116023	5
10118835	5
15004834	5
15005345	5
10000991	4
10002437	4
10003430	4
10004890	4
10005766	4
10006844	4
10007354	4
10013313	4
10013565	4
10014683	4
10014977	4
10015646	4
10015863	4
10015888	4
10016910	4
10019616	4
10021762	4
10021861	4
10022783	4
10024215	4
10025035	4
10025274	4
10026785	4
10027203	4
10027977	4
10028192	4
10028443	4
10030319	4
[illegible]	4

Tabulate

DiagnIBUIFinal	N
10032976	4
10038998	4
10040549	4
10040817	4
10043305	4
10045942	4
10048512	4
10049031	4
10049272	4
10049824	4
10052176	4
10055550	4
10059021	4
10072167	4
10072412	4
10073459	4
10073615	4
10075479	4
10076853	4
10080406	4
10080468	4
10080554	4
10080682	4
10080686	4
10080710	4
10081834	4
10082065	4
10085496	4
10087342	4
10091384	4
10092181	4
10092408	4
10097842	4
10105600	4
10105714	4
10107132	4
10107421	4
10108925	4
10109130	4
10114322	4
10114469	4
15000843	4
15004306	4
15005323	4
10000549	3
10000900	3
10001010	3
10002432	3
[illegible]	[illegible]

Tabulate

DiagnIBUIFinal	N
10002529	3
10004709	3
10004763	3
10006289	3
10006431	3
10007684	3
10008184	3
10008186	3
10008661	3
10009583	3
10010774	3
10011161	3
10011892	3
10013321	3
10013524	3
10014696	3
10015222	3
10018004	3
10022145	3
10026583	3
10026614	3
10026703	3
10028003	3
10028920	3
10030968	3
10031569	3
10031814	3
10033328	3
10033338	3
10034202	3
10034634	3
10036474	3
10036504	3
10038979	3
10039586	3
10039815	3
10039825	3
10040168	3
10041354	3
10041557	3
10044508	3
10045286	3
10045966	3
10046494	3
10048169	3
10050031	3
10050563	3
10051009	3
[illegible]	[illegible]

Tabulate

DiagnIBUIFinal	N
10058365	3
10059500	3
10059540	3
10060310	3
10060657	3
10063184	3
10071163	3
10071374	3
10073742	3
10074473	3
10074485	3
10074786	3
10075135	3
10075168	3
10076197	3
10077303	3
10077318	3
10077690	3
10077691	3
10078497	3
10079930	3
10081619	3
10084322	3
10084839	3
10085737	3
10087268	3
10089874	3
10090651	3
10096158	3
10096490	3
10096985	3
10097294	3
10097808	3
10099395	3
10101321	3
10102048	3
10102412	3
10104797	3
10106122	3
10106181	3
10107089	3
10107442	3
10108037	3
10111013	3
10117960	3
10118034	3
10122930	3
15001537	3
[illegible]	[illegible]

Tabulate

DiagnIBUIFinal	N
15001928	3
15002159	3
15002789	3
15003082	3
15003689	3
15003983	3
15004078	3
15004933	3
15005023	3
15005282	3
10000296	2
10000552	2
10000920	2
10000990	2
10001093	2
10001564	2
10002097	2
10002440	2
10002492	2
10003441	2
10003529	2
10004012	2
10004688	2
10004837	2
10007488	2
10007609	2
10008257	2
10009695	2
10011144	2
10011906	2
10012030	2
10013031	2
10013099	2
10015642	2
10015884	2
10016278	2
10016902	2
10019298	2
10020802	2
10021408	2
10021697	2
10021768	2
10021867	2
10021981	2
10022144	2
10022199	2
10022330	2
10022478	2
[illegible]	[illegible]

Tabulate

DiagnIBUIFinal	N
10024020	2
10025333	2
10025457	2
10026658	2
10027220	2
10027238	2
10027591	2
10027617	2
10027837	2
10027908	2
10028175	2
10028528	2
10030002	2
10030999	2
10031499	2
10031537	2
10031568	2
10032899	2
10034125	2
10034234	2
10034350	2
10034950	2
10037107	2
10037321	2
10037551	2
10037699	2
10037729	2
10038396	2
10038484	2
10038970	2
10039427	2
10039912	2
10039937	2
10040038	2
10040493	2
10041071	2
10041738	2
10042036	2
10042054	2
10043017	2
10043296	2
10044595	2
10044931	2
10045168	2
10045421	2
10045432	2
10045482	2
10045529	2
[illegible]	[illegible]

Tabulate

DiagnIBUIFinal	N
10045711	2
10045903	2
10046504	2
10046735	2
10047038	2
10047766	2
10048632	2
10049087	2
10049774	2
10049777	2
10049906	2
10050596	2
10051605	2
10052352	2
10052492	2
10056133	2
10056806	2
10058545	2
10059584	2
10060311	2
10061534	2
10061568	2
10062136	2
10063197	2
10071635	2
10071940	2
10071977	2
10074794	2
10074805	2
10074836	2
10075349	2
10075363	2
10075504	2
10075506	2
10075569	2
10075592	2
10076860	2
10077069	2
10077238	2
10078530	2
10078587	2
10078925	2
10079075	2
10079129	2
10079324	2
10079450	2
10079544	2
10080184	2
[illegible]	[illegible]

Tabulate

DiagnIBUIFinal	N
10080326	2
10080465	2
10080685	2
10080865	2
10081285	2
10083136	2
10083545	2
10083946	2
10084099	2
10084320	2
10085260	2
10086860	2
10087595	2
10088929	2
10090987	2
10091032	2
10091166	2
10091362	2
10091672	2
10092268	2
10093824	2
10094556	2
10097007	2
10098095	2
10098203	2
10099202	2
10099549	2
10101414	2
10102175	2
10102421	2
10103304	2
10104087	2
10104964	2
10105593	2
10105613	2
10105707	2
10105973	2
10105975	2
10106864	2
10107646	2
10108757	2
10110508	2
10110637	2
10110965	2
10111114	2
10111181	2
10111305	2
10111329	2
[illegible]	[illegible]

Tabulate

DiagnIBUIFinal	N
10111460	2
10111746	2
10111972	2
10112164	2
10117450	2
10118687	2
10119717	2
10121339	2
10123312	2
10125172	2
10125219	2
10125289	2
10125293	2
15001351	2
15003514	2
15003868	2
15004005	2
15004098	2
15004173	2
15004882	2
15004915	2
15004934	2
15004955	2
15005045	2
15005088	2
15005341	2
20000001	2
20000002	2
10000065	1
10000094	1
10000225	1
10000257	1
10000407	1
10000509	1
10000516	1
10000532	1
10000699	1
10000730	1
10000750	1
10000751	1
10000866	1
10000917	1
10000933	1
10000950	1
10000956	1
10000958	1
10000970	1
10000973	1
[illegible]	[illegible]

Tabulate

DiagnIBUIFinal	N
10001004	1
10001105	1
10001141	1
10001199	1
10001210	1
10001966	1
10002019	1
10002023	1
10002024	1
10002164	1
10002177	1
10002287	1
10002434	1
10002633	1
10002686	1
10002900	1
10003133	1
10003177	1
10003322	1
10003475	1
10004011	1
10004542	1
10004666	1
10004819	1
10005163	1
10005457	1
10005556	1
10006115	1
10006236	1
10006246	1
10006251	1
10006269	1
10006277	1
10006381	1
10006425	1
10006599	1
10006830	1
10006835	1
10006837	1
10007632	1
10007639	1
10007650	1
10007689	1
10008122	1
10008198	1
10008200	1
10008471	1
10008540	1
[illegible]	[illegible]

Tabulate

DiagnIBUIFinal	N
10008664	1
10008852	1
10008873	1
10009043	1
10009179	1
10009293	1
10009367	1
10009374	1
10009376	1
10009392	1
10009403	1
10009559	1
10009696	1
10009934	1
10009936	1
10010166	1
10010198	1
10010213	1
10010215	1
10010276	1
10010379	1
10010545	1
10010654	1
10011072	1
10011333	1
10011376	1
10011379	1
10011761	1
10011887	1
10011914	1
10012242	1
10012649	1
10012668	1
10012742	1
10012952	1
10012961	1
10013173	1
10013175	1
10013186	1
10013264	1
10013304	1
10013423	1
10013480	1
10013487	1
10013504	1
10013626	1
10013806	1
10014031	1
10014631	1

Tabulate

DiagnIBUIFinal	N
10014631	1
10014767	1
10014896	1
10015146	1
10015258	1
10015287	1
10015369	1
10015422	1
10015640	1
10015669	1
10015674	1
10015709	1
10015788	1
10015797	1
10015799	1
10015871	1
10015875	1
10015889	1
10015894	1
10015913	1
10016298	1
10016930	1
10016969	1
10017386	1
10017594	1
10017619	1
10017788	1
10017789	1
10017937	1
10017969	1
10017977	1
10017981	1
10018322	1
10018385	1
10018553	1
10018589	1
10018648	1
10018650	1
10018651	1
10019016	1
10019090	1
10019207	1
10019286	1
10019413	1
10019414	1
10019501	1
10019526	1
10019610	1
[illegible]	[illegible]

Tabulate

DiagnIBUIFinal	N
10019747	1
10019874	1
10019905	1
10019936	1
10020967	1
10021257	1
10021380	1
10021390	1
10021538	1
10021614	1
10021763	1
10021840	1
10021863	1
10021871	1
10021897	1
10021970	1
10021975	1
10022005	1
10022275	1
10022281	1
10022298	1
10022414	1
10022459	1
10022469	1
10022507	1
10022735	1
10023006	1
10023093	1
10023136	1
10023265	1
10023297	1
10023304	1
10023307	1
10023325	1
10023488	1
10023515	1
10023590	1
10023647	1
10023892	1
10024041	1
10024252	1
10024380	1
10024833	1
10025270	1
10025435	1
10025768	1
10025858	1
10026283	1
[illegible]	[illegible]

Tabulate

DiagnIBUIFinal	N
10026418	1
10026526	1
10026527	1
10026531	1
10026542	1
10026556	1
10026561	1
10026563	1
10026623	1
10026634	1
10026795	1
10027158	1
10027159	1
10027201	1
10027204	1
10027235	1
10027334	1
10027568	1
10027899	1
10027911	1
10027945	1
10028197	1
10028525	1
10028590	1
10028627	1
10028782	1
10028861	1
10028875	1
10029188	1
10029888	1
10030039	1
10030347	1
10030474	1
10030489	1
10030602	1
10030725	1
10030728	1
10030734	1
10030819	1
10030840	1
10030881	1
10030915	1
10030966	1
10031019	1
10031123	1
10031146	1
10031371	1
10031493	1
[illegible]	[illegible]

Tabulate

DiagnIBUIFinal	N
10031578	1
10031580	1
10031860	1
10031875	1
10032025	1
10032027	1
10032201	1
10032234	1
10032278	1
10032307	1
10032397	1
10032561	1
10032880	1
10033413	1
10033454	1
10033715	1
10033944	1
10034128	1
10034152	1
10034179	1
10034407	1
10034752	1
10034812	1
10035197	1
10035233	1
10035236	1
10035266	1
10035304	1
10035562	1
10035668	1
10036103	1
10036494	1
10036535	1
10036748	1
10037243	1
10037751	1
10037804	1
10038312	1
10038409	1
10038466	1
10038873	1
10039012	1
10039325	1
10039364	1
10039365	1
10039371	1
10039434	1
10039537	1
[illegible]	[illegible]

Tabulate

DiagnIBUIFinal	N
10039580	1
10039589	1
10039643	1
10039787	1
10039842	1
10040099	1
10040223	1
10040263	1
10040341	1
10040818	1
10040872	1
10041129	1
10041476	1
10041533	1
10041788	1
10041810	1
10041873	1
10041875	1
10041902	1
10042046	1
10042092	1
10042577	1
10042584	1
10043040	1
10043225	1
10043400	1
10044172	1
10044470	1
10044624	1
10044665	1
10044690	1
10044970	1
10045141	1
10045391	1
10045627	1
10045657	1
10045659	1
10045703	1
10045746	1
10045829	1
10045959	1
10045976	1
10045981	1
10046419	1
10046445	1
10046752	1
10047473	1
10047566	1
[illegible]	[illegible]

Tabulate

DiagnIBUIFinal	N
10047719	1
10047733	1
10047737	1
10047749	1
10047758	1
10048053	1
10048260	1
10048325	1
10048360	1
10048378	1
10048440	1
10048500	1
10048796	1
10049076	1
10049129	1
10049510	1
10049818	1
10050301	1
10050565	1
10050705	1
10051033	1
10051619	1
10051676	1
10051682	1
10051776	1
10052256	1
10052456	1
10052549	1
10052608	1
10052668	1
10052860	1
10053401	1
10053549	1
10053715	1
10053763	1
10053920	1
10055010	1
10055227	1
10055588	1
10055694	1
10055704	1
10055710	1
10056008	1
10056009	1
10056270	1
10056366	1
10057496	1
10057503	1
[illegible]	[illegible]

Tabulate

DiagnIBUIFinal	N
10057569	1
10057974	1
10058550	1
10059020	1
10059125	1
10060075	1
10060196	1
10060368	1
10060653	1
10061372	1
10061436	1
10061558	1
10061567	1
10061636	1
10061817	1
10062229	1
10062846	1
10062946	1
10063230	1
10071091	1
10071101	1
10071125	1
10071381	1
10071390	1
10071668	1
10071674	1
10071770	1
10072177	1
10072555	1
10072599	1
10072601	1
10072741	1
10072812	1
10073417	1
10073558	1
10074011	1
10074157	1
10074468	1
10074796	1
10074807	1
10074832	1
10074844	1
10074939	1
10075422	1
10075445	1
10075468	1
10075476	1
10075565	1
[illegible]	[illegible]

Tabulate

DiagnIBUIFinal	N
10075830	1
10075932	1
10075983	1
10076666	1
10076854	1
10076984	1
10077373	1
10077390	1
10077645	1
10078195	1
10078262	1
10078564	1
10078607	1
10078900	1
10079008	1
10079068	1
10079162	1
10079396	1
10080047	1
10080048	1
10080173	1
10080552	1
10080556	1
10080557	1
10080558	1
10080660	1
10080675	1
10080729	1
10080793	1
10081247	1
10081458	1
10081830	1
10081953	1
10082068	1
10082541	1
10082719	1
10082775	1
10083131	1
10083470	1
10083585	1
10083733	1
10083943	1
10084334	1
10084375	1
10084631	1
10084907	1
10085041	1
10085197	1
[illegible]	1

Tabulate

DiagnIBUIFinal	N
10085198	1
10085208	1
10085768	1
10085806	1
10085841	1
10085915	1
10086344	1
10086404	1
10086518	1
10087310	1
10087508	1
10087626	1
10087909	1
10088050	1
10088364	1
10088506	1
10088551	1
10088558	1
10088599	1
10088889	1
10089096	1
10089265	1
10089275	1
10089483	1
10089566	1
10090349	1
10090382	1
10090449	1
10090600	1
10090602	1
10090868	1
10090951	1
10090990	1
10091055	1
10091058	1
10091167	1
10091194	1
10091499	1
10091549	1
10091658	1
10091668	1
10091689	1
10091949	1
10092164	1
10092180	1
10092269	1
10092270	1
10092346	1
[illegible]	1

Tabulate

DiagnIBUIFinal	N
10092372	1
10092411	1
10092664	1
10093182	1
10093436	1
10094128	1
10094164	1
10094319	1
10096128	1
10096262	1
10096510	1
10096572	1
10096893	1
10096925	1
10096934	1
10096937	1
10096989	1
10097016	1
10097059	1
10097268	1
10097350	1
10097489	1
10099113	1
10099203	1
10099399	1
10099596	1
10099619	1
10099960	1
10101421	1
10101548	1
10101785	1
10102266	1
10102267	1
10102272	1
10103007	1
10103160	1
10103337	1
10103518	1
10103784	1
10104049	1
10104117	1
10104528	1
10104555	1
10104594	1
10104810	1
10104913	1
10104919	1
10105114	1
[illegible]	1

Tabulate

DiagnIBUIFinal	N
10105430	1
10105543	1
10105717	1
10106001	1
10106055	1
10106207	1
10106220	1
10106485	1
10106934	1
10107243	1
10107444	1
10107563	1
10107609	1
10107622	1
10107650	1
10107664	1
10107672	1
10107774	1
10107793	1
10108056	1
10108361	1
10108679	1
10108913	1
10108956	1
10109119	1
10109418	1
10110253	1
10110296	1
10110319	1
10110331	1
10110875	1
10111021	1
10111023	1
10111089	1
10111332	1
10111369	1
10111380	1
10111444	1
10111575	1
10111698	1
10111825	1
10111918	1
10111953	1
10111956	1
10112518	1
10112532	1
10113037	1
10113600	1
10113681	1

Tabulate

DiagnIBUIFinal	N
10113681	1
10114220	1
10114357	1
10114538	1
10114880	1
10115166	1
10115217	1
10115261	1
10115959	1
10116049	1
10116054	1
10116216	1
10117301	1
10117451	1
10117509	1
10117866	1
10118022	1
10118035	1
10118107	1
10118490	1
10118678	1
10118760	1
10118792	1
10118860	1
10119725	1
10120032	1
10121154	1
10121191	1
10121273	1
10121278	1
10121282	1
10121317	1
10121375	1
10121646	1
10121979	1
10121980	1
10122200	1
10122249	1
10123033	1
10123398	1
10123680	1
10124190	1
10124286	1
10124839	1
15000479	1
15000918	1
15000966	1
15001338	1
[illegible]	1

Tabulate

DiagnIBUIFinal	N
15001377	1
15001584	1
15001597	1
15001902	1
15001920	1
15001927	1
15001994	1
15002176	1
15002284	1
15002285	1
15002371	1
15002880	1
15003020	1
15003734	1
15003872	1
15003911	1
15004026	1
15004142	1
15004203	1
15004241	1
15004282	1
15004320	1
15004328	1
15004347	1
15004348	1
15004394	1
15004803	1
15004837	1
15004876	1
15004911	1
15004937	1
15004939	1
15005058	1
15005071	1
15005086	1
15005115	1
15005116	1
15005242	1
15005273	1
15005277	1
15005315	1
15005317	1
15005325	1
15005326	1
15005329	1
20000012	1
20000019	1
20000039	1

Tabulate

DiagnIBUIFinal	N

8322 rows have been excluded.

werkbriefjes

Tabulate

	huisbezoek					
	Year					
	2019			2020		
ContArbeidsongeschikt	Column %	N	Row %	Column %	N	Row %
0	97,97%	1109	5,39%	98,80%	1403	6,81%
1	2,03%	23	0,87%	1,20%	17	0,64%
All	100,00%	1132	4,87%	100,00%	1420	6,11%

751 rows have been excluded.

minder werkbriefjes nodig met de tijd?

Local Data Filter

15655 matching rows

☐ Inverse

Year (2)

2019 2020

Tabulate

Year = 2020

	ContArbeidsongeschikt					
	0		1		All	
WeekendNr	Row %	N	Row %	N	Row %	N
1	88,16%	3142	11,84%	422	100,00%	3564
2	89,50%	3249	10,50%	381	100,00%	3630
3	92,59%	2688	7,41%	215	100,00%	2903
4	92,60%	3343	7,40%	267	100,00%	3610
5	92,15%	1795	7,85%	153	100,00%	1948
All	90,81%	14217	9,19%	1438	100,00%	15655

8322 rows have been excluded.

Verwijzingen

Tabulate

	Year									
	2019				2020					
	ConsultType				ConsultType					
	huisbezoek		raadpleging		huisbezoek		raadpleging		telefonisch consult	
EDReferal	N	Column %	N	Column %	N	Column %	N	Column %	N	Column %
0	989	87,37%	6101	94,75%	1260	88,73%	4815	90,66%	8712	97,62%
1	143	12,63%	338	5,25%	160	11,27%	496	9,34%	212	2,38%

751 rows have been excluded.

ConsultType											
raadpleging						telefonisch consult			All		
Year						Year					
2019			2020			2020					
Column %	N	Row %	Column %	N	Row %	Column %	N	Row %	Column %	N	Row %
81,72%	5262	25,56%	87,54%	4649	22,58%	91,49%	8165	39,66%	88,64%	20588	100,00%
18,28%	1177	44,62%	12,46%	662	25,09%	8,51%	759	28,77%	11,36%	2638	100,00%
100,00%	6439	27,72%	100,00%	5311	22,87%	100,00%	8924	38,42%	100,00%	23226	100,00%

Local Data Filter

8808 matching rows

☐ Inverse

SuspectedCovid 2 (3)

. | 0 | 1

Tabulate

SuspectedCovid 2 = 1

	Year			
	2019			
	ConsultType			
	huisbezoek		raadpleging	
EDReferal	N	Column %	N	Column %
0	188	89,52%	1638	98,14%
1	22	10,48%	31	1,86%

15406 rows have been excluded.

RFE bij suspected covid

Local Data Filter

6692 matching rows

☐ Inverse

SuspectedCovid 2 (3)

. | 0 | 1

Year (2)

2019 | 2020

Tabulate

(SuspectedCovid 2 = 1) and (Year = 2020)

ContactRedenTekst	N
Koorts	1665
Hoesten	1351
Kortademigheid	711
Influenza	680
Symptomen/klachten keel	596
Andere virusziekte/virusziekte nao	315
Acute infectie bovenste luchtwegen	141
Algemene moeheid/zwakte	123
Diarree	118
Pijn op de borst nao	115
Hoofdpijn	82
Zich ziek voelen	73
Angst voor andere ziekte luchtwegen	71
Spierpijn	61
Ander probleem ademhaling	36
Angststoornis/angsttoestand	36
Braken	35
Niezen/neusverstopping	29
Andere infectieziekte nao	26
Gegeneraliseerde buikpijn/buikkrampen	26
Andere infectie luchtwegen	24
Stoornis reuk/smaak	23
Hartkloppingen/bewust van hartslag	19
Oorpijn	18
Ziekte/toestand van niet-gespecificeerde aard/lokalisatie	18
Misselijkheid	17
Pijn toegeschreven aan luchtwegen	17
Vertigo/duizeligheid	15
Dood	12
Gegeneraliseerde pijn/pijn multipele plaatsen	12
[illegible]	[illegible]

2020					
ConsultType					
huisbezoek		raadpleging		telefonisch consult	
N	Column %	N	Column %	N	Column %
287	84,91%	1319	90,34%	4798	98,04%
51	15,09%	141	9,66%	96	1,96%

Tabulate

ContactRedenTekst	N
Druk/beklemming toegeschreven aan hart	11
Symptomen/klachten stem	11
Allergische rhinitis	10
Maagpijn	9
Symptomen/klachten rug	9
Andere ziekte luchtwegen	8
Geheugenstoornis	7
Koude rillingen	7
Pijn oog	7
Rood oog	7
Acute bronchitis/bronchiolitis	5
Acute/chronische sinusitis	5
Astma	5
Flauwvallen/syncope	5
Gastro-intestinale infectie	5
Haemoptoe	5
Pneumonie	5
Allergie/allergische reactie nao	4
Andere algemene symptomen/klachten	4
Geen ziekte	4
Hypertensie zonder orgaanbeschadiging	4
Lokaal exantheem	4
Piepende ademhaling	4
Ander letsel oog	3
Chronisch obstructieve longziekte (COPD)	3
Hyperventilatiesyndroom	3
Orthostatische hypotensie	3
Preventie	3
Verlamming/krachtsverlies	3
Andere symptomen/klachten luchtwegen	2
Andere symptomen/klachten neus	2
Epistaxis/neusbloeding	2
Furunkel/abces neus	2
Jeuk	2
Medicatie - recept/verzoek/herhaling/injectie	2
Pijnlijke mictie	2
Prikkelbaar/boos gevoel/gedrag	2
Symptomen/klachten been/dijbeen	2
Symptomen/klachten borstkas	2
Symptomen/klachten mond/tong/lippen	2
Abnormaal uiterlijk oog	1
Abnormale onwillekeurige bewegingen	1
Ander letsel bewegingsapparaat	1
Andere symptomen/klachten zenuwstelsel	1
Andere ziekte bewegingsapparaat	1
Andere ziekte oog/oogadnexen	1
Andere/niet gespecificeerde anemie	1
Angst voor andere ziekte nao	1
[illegible]	[illegible]

Tabulate

ContactRedenTekst	N
Cataract	1
Chronische/niet-specifieke lymphadenitis	1
Coma	1
Complicatie medische behandeling	1
Corpus alienum neus/larynx/bronchus	1
Dementie	1
Doofheid	1
Gezwollen enkels/enkeloedeem	1
Hematurie	1
Hypoglykemie	1
Ischemische hartziekte met angina	1
Lokale zwelling/knobbel huid	1
Melaena	1
Migraine	1
Overmatig cerumen	1
Pijn cardiovasculair stelsel nao	1
Pijn toegeschreven aan hart	1
Pleuritis/pleuravocht	1
Prikkelbare zuigeling	1
Probleem met ziek zijn/acceptatie ziekte	1
Psoriasis	1
Risicofactor nao	1
Slikprobleem	1
Symptomen/klachten nek	1
Tintelen vingers/voeten/tenen	1
Verandering in kleur huid	1
Vergrote/pijnlijke lymfeklier(en)	1
Verminderde eetlust	1
Zich oud voelen/gedragen	1
Ziekte mond/tong/lippen	1
Ziekte oesophagus	1
Zuurbranden	1
Zwangerschap	1
Zweetprobleem	1

17285 rows have been excluded.

diagnosis bij suspected covid

Local Data Filter

6692 matching rows

☐ Inverse

Year (2)

2019 | 2020

SuspectedCovid 2 (3)

. | 0 | 1

Tabulate

(Year = 2020) and (SuspectedCovid 2 = 1)

DiagnFinalLabel
Coronavirusinfectie: mogelijk geval COVID19
koorts
hoesten
niet gespecificeerd viraal syndroom
Angst voor niet aangetoonde andere ziekte luchtwegen (bv. COVID19 coronavirusinfe
[illegible]

	N	Column %
	3131	46,79%
	599	8,95%
	365	5,45%
	317	4,74%
ectie)	254	3,80%
	[illegible]	[illegible]

Local Data Filter

Tabulate

DiagnFinalLabel
keelpijn
niet gespecificeerde virale infectie
niet gespecificeerde acute bovenste luchtweginfectie
diarree
griep
niet gespecificeerde virale bovenste luchtweginfectie
niet gespecificeerde bronchitis
coronavirus infectie
kortademigheid
niet gespecificeerde pneumonie
niet gespecificeerde bovenste luchtwegontsteking
niet gespecificeerde pharyngitis
verkoudheid
niet gespecificeerde keelontsteking
bovenste luchtwegaandoening
droge hoest
virale verkoudheid
Coronavirusinfectie: nauw contact met coronavirus zonder actuele klinische verdenkin
vermoeidheid
andere luchtwegeninfectie
hooikoorts
acute onderste luchtweginfectie
niet gespecificeerde acute bronchitis
rhinitis
niet gespecificeerde keelinfectie
niet gespecificeerde acute keelpijn
moe
virale keelpijn
algehele lichamelijke achteruitgang
griep verkoudheid
allergische rhinitis
acute COPD exacerbatie
andere conjunctivitis
hyperventilatie dyspnoe
postnasale drip
tracheïtis
virale pharyngitis
anosmie
influenza
prikkelhoest
rhinopharyngitis
BHR bronchiale hyperreactiviteit
niet gespecificeerde virale aandoening
ademhalingspijn
ademnood
asthenie
bronchopneumonie
griep enteritis
[illegible]

	N	Column %
	223	3,33%
	206	3,08%
	133	1,99%
	106	1,58%
	106	1,58%
	100	1,49%
	91	1,36%
	86	1,29%
	76	1,14%
	75	1,12%
	61	0,91%
	53	0,79%
	45	0,67%
	40	0,60%
	37	0,55%
	35	0,52%
	35	0,52%
ıg voor COVID19	29	0,43%
	25	0,37%
	24	0,36%
	20	0,30%
	18	0,27%
	18	0,27%
	18	0,27%
	17	0,25%
	14	0,21%
	13	0,19%
	12	0,18%
	11	0,16%
	11	0,16%
	10	0,15%
	9	0,13%
	9	0,13%
	9	0,13%
	9	0,13%
	9	0,13%
	9	0,13%
	8	0,12%
	8	0,12%
	8	0,12%
	8	0,12%
	7	0,10%
	7	0,10%
	6	0,09%
	6	0,09%
	6	0,09%
	6	0,09%
	6	0,09%
	[illegible]	[illegible]

Tabulate

DiagnFinalLabel
neusverkoudheid
niet gespecificeerde virale conjunctivitis
conjunctiva oedeem
griep met keelpijn
griepbronchitis
loopneus
neusverstopping
niet gespecificeerde virale pneumonie
productieve hoest
valse kroep
virale bronchitis
zwak
COPD met acute exacerbatie
acute rhinitis
bloeding bij hoesten
bronchiaal hoesten
keelabces
laryngitis
orthopnoe
tachypnoe
abnormale ademhaling
febris e.c.i.
haemoptoe
lokale neusinfectie
neusobstructie
niet gespecificeerde acute bronchiolitis
niet gespecificeerde longontsteking
niet gespecificeerde viremie
pollenallergie
rhinovirus luchtweginfectie
smaakzinstoornis
verstopte neus
andere virale pneumonie
angina aphthosa
astmarhinitis
blefaroconjunctivitis
bloederig sputum
borstpijn bij ademhaling
bronchiaal hoesten met griep of influenza
bronchusspasme
chronisch hoesten
chronische bronchitis
coryza
dunne ontlasting
epidemisch hoesten
epidermale allergie
grieppharyngitis
grieppneumonie
[illegible]

	N	Column %
	5	0,07%
	5	0,07%
	4	0,06%
	4	0,06%
	4	0,06%
	4	0,06%
	4	0,06%
	4	0,06%
	4	0,06%
	4	0,06%
	4	0,06%
	4	0,06%
	3	0,04%
	3	0,04%
	3	0,04%
	3	0,04%
	3	0,04%
	3	0,04%
	3	0,04%
	3	0,04%
	2	0,03%
	2	0,03%
	2	0,03%
	2	0,03%
	2	0,03%
	2	0,03%
	2	0,03%
	2	0,03%
	2	0,03%
	2	0,03%
	2	0,03%
	2	0,03%
	1	0,01%
	1	0,01%
	1	0,01%
	1	0,01%
	1	0,01%
	1	0,01%
	1	0,01%
	1	0,01%
	1	0,01%
	1	0,01%
	1	0,01%
	1	0,01%
	1	0,01%
	1	0,01%
	1	0,01%
	1	0,01%
	1	0,01%

Tabulate

DiagnFinalLabel
hoge temperatuur
hooikoortscatarre
hooikoortscatarre met astma
influenza luchtweg infectie
influenza verkoudheid
influenza-achtige ziekte
inspiratoir piepen
irregulaire ademhaling
keelaandoening
kroep
laryngitis stridulosa
lethargie
longaandoening bij elders geclassificeerde ziekten
longcyste
longinfectie
membraneuse angina
moeizame ademhaling
niet gespecificeerd keelulcus
niet gespecificeerde acute adenoïditis
niet gespecificeerde acute of subacute tonsilinfectie
niet gespecificeerde farynxinfectie
pharyngotracheïtis
pleuritische pijn
pollinose
respiratoire distress
respiratoire zwakte of insufficiëntie
rhinorroe
rinorroe
slijmaspiratie in keel
smaakzinverlies
snelle ademhaling
tonsilabces
volledige anosmie
zwakke ademhaling

17285 rows have been excluded.

	N	Column %
	1	0,01%
	1	0,01%
	1	0,01%
	1	0,01%
	1	0,01%
	1	0,01%
	1	0,01%
	1	0,01%
	1	0,01%
	1	0,01%
	1	0,01%
	1	0,01%
	1	0,01%
	1	0,01%
	1	0,01%
	1	0,01%
	1	0,01%
	1	0,01%
	1	0,01%
	1	0,01%
	1	0,01%
	1	0,01%
	1	0,01%
	1	0,01%
	1	0,01%
	1	0,01%
	1	0,01%
	1	0,01%
	1	0,01%
	1	0,01%
	1	0,01%
	1	0,01%
	1	0,01%
	1	0,01%

Local Data Filter

6692 matching rows

☐ Inverse

Year (2)

2019 | 2020

SuspectedCovid 2 (3)

. | 0 | 1

Tabulate

(Year = 2020) and (SuspectedCovid 2 = 1)

DiagnICPCTitelFinal	N	Column %
Influenza	3275	48,94%
Andere virusziekte nao	618	9,23%
Koorts	602	9,00%
Acute infectie bovenste luchtwegen	546	8,16%
Hoesten	417	6,23%
Angst voor andere ziekte luchtwegen	283	4,23%
Symptomen/klachten keel	223	3,33%
Acute bronchitis/bronchiolitis	133	1,99%
Diarree	107	1,60%
Kortademigheid	89	1,33%
Pneumonie	89	1,33%
Andere ziekte luchtwegen	73	1,09%
Algemene moeheid/zwakte	60	0,90%
Allergische rhinitis	35	0,52%
Andere infectie luchtwegen	19	0,28%
Ander probleem ademhaling	16	0,24%
Infectieuze conjunctivitis	15	0,22%
Acute laryngitis/tracheitis	14	0,21%
Chronisch obstructieve longziekte	12	0,18%
Stoornis reuk/smaak	12	0,18%
Andere symptomen/klachten neus	9	0,13%
Astma	9	0,13%
Niezen/neusverstopping	9	0,13%
Pijn toegeschreven aan luchtwegen	8	0,12%
Haemoptoe	6	0,09%
Andere ziekte oog/oogadnexen	4	0,06%
Acute tonsillitis	3	0,04%
Furunkel/abces neus	2	0,03%
Chronische bronchitis	1	0,01%
Corpus alienum neus/larynx/bronchus	1	0,01%
Hyperventilatiesyndroom	1	0,01%
Piepende ademhaling	1	0,01%

17285 rows have been excluded.

Local Data Filter

6692 matching rows

☐ Inverse

Year (2)

2019 | 2020

SuspectedCovid 2 (3)

. | 0 | 1

Tabulate

(Year = 2020) and (SuspectedCovid 2 = 1)

DiagnICPCchapter	N	Column %
R	5274	78,81%
A	1280	19,13%
D	107	1,60%
F	19	0,28%
N	12	0,18%

17285 rows have been excluded.

verwwijzingen onder suspected covids

Local Data Filter

6692 matching rows

☐ Inverse

SuspectedCovid 2 (3)

. | 0 | 1

Year (2)

2019 | 2020

Tabulate

(SuspectedCovid 2 = 1) and (Year = 2020)

	ConsultType					
	huisbezoek		raadpleging		telefonisch consult	
EDReferal	N	Column %	N	Column %	N	Column %
0	287	84,91%	1319	90,34%	4798	98,04%
1	51	15,09%	141	9,66%	96	1,96%
All	338	100,00%	1460	100,00%	4894	100,00%

17285 rows have been excluded.

ICPC chapters

Local Data Filter

12477 matching rows

☐ Inverse

ConsultType (3)

huisbezoek (2575)

raadpleging (12477)

telefonisch consult (8925)

Tabulate

	Drop zone for columns
Drop zone for rows	Resulting Cells

All	
N	Column %
6404	95,70%
288	4,30%
6692	100,00%

Local Data Filter

15655 matching rows

☐ Inverse

Year (2)

2019 2020

Tabulate

Year = 2020

	SuspectedCovid 2			
	0		1	
WeekendNr	N	Row %	N	Row %
1	1509	42,34%	2055	57,66%
2	1791	49,34%	1839	50,66%
3	1721	59,28%	1182	40,72%
4	2527	70,00%	1083	30,00%
5	1415	72,64%	533	27,36%

8322 rows have been excluded.
