# Supplementary material for: Organisation and characteristics of out-of-hours primary care during a COVID-19 outbreak: A real-time observational study
Source: PLoS One. 2020 Aug 13;15(8):e0237629. doi: 10.1371/journal.pone.0237629 (PMC7425859; doi:10.1371/journal.pone.0237629)
Supplement: S2 File — (PDF) [file pone.0237629.s003.pdf]

Local Data Filter

15655 matching rows

☐ Inverse

Year (2)

2019 | 2020

Contingency Analysis of ConsultType By Geslacht

Mosaic Plot

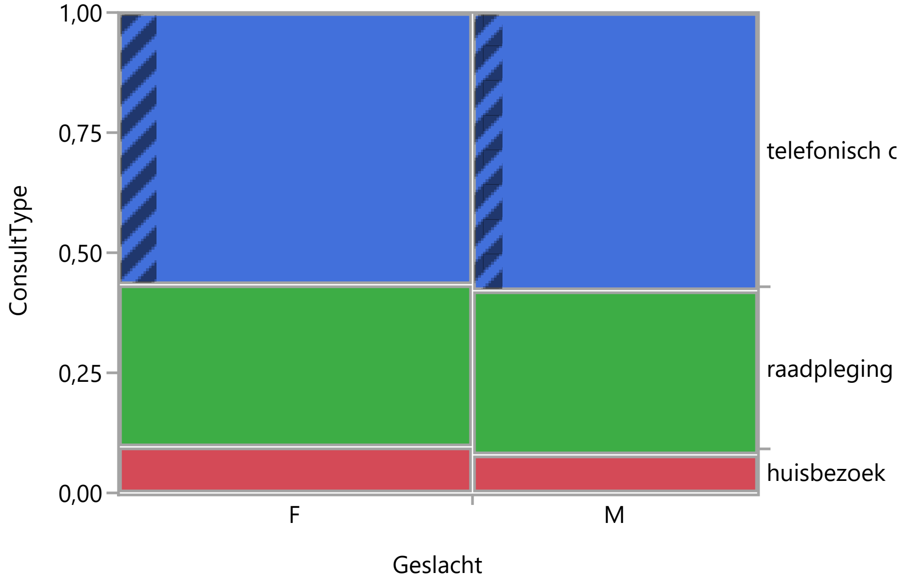

Contingency Table

ConsultType

Geslacht	Count / Col %	huisbezoek	raadpleging	telefonisch consult	Total
	F	856	2921	4894	8671
		60,28	55,00	54,84	
	M	564	2390	4030	6984
		39,72	45,00	45,16	
	Total	1420	5311	8924	15655

Tests

N	DF	-LogLike	RSquare (U)
15655	2	7,6447343	0,0005

Test	ChiSquare	Prob>ChiSq
Likelihood Ratio	15,289	0,0005*
Pearson	15,169	0,0005*

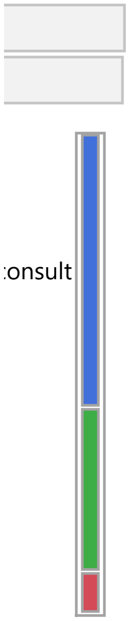
consult

Local Data Filter

15655 matching rows

☐ Inverse

Year (2)

2019 | 2020

Contingency Analysis of ConsultType By SuspectedCovid 2

Mosaic Plot

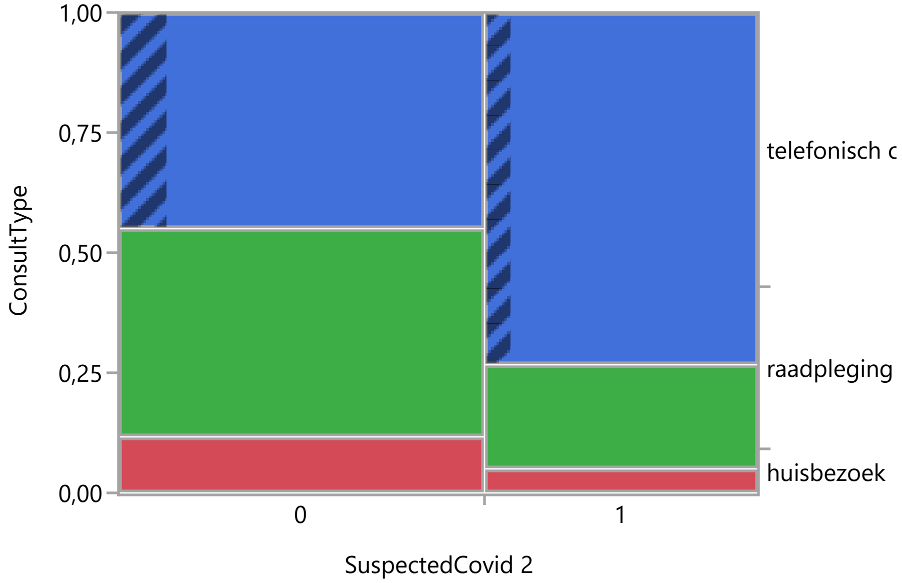

Contingency Table

ConsultType (columns) / SuspectedCovid 2 (rows)

Count<br>Col %	huisbezoek	raadpleging	telefonisch consult	Total
0	1082<br>76,20	3851<br>72,51	4030<br>45,16	8963
1	338<br>23,80	1460<br>27,49	4894<br>54,84	6692
Total	1420	5311	8924	15655

Tests

N	DF	-LogLike	RSquare (U)
15655	2	639,61381	0,0452

Test	ChiSquare	Prob>ChiSq
Likelihood Ratio	1279,228	<,0001*
Pearson	1246,679	<,0001*

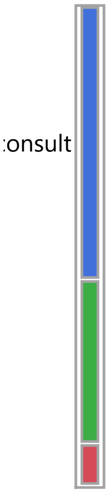

Local Data Filter

15655 matching rows

☐ Inverse

Year (2)

2019 | 2020

Contingency Analysis of ConsultType By ContArbeidsongeschikt

Mosaic Plot

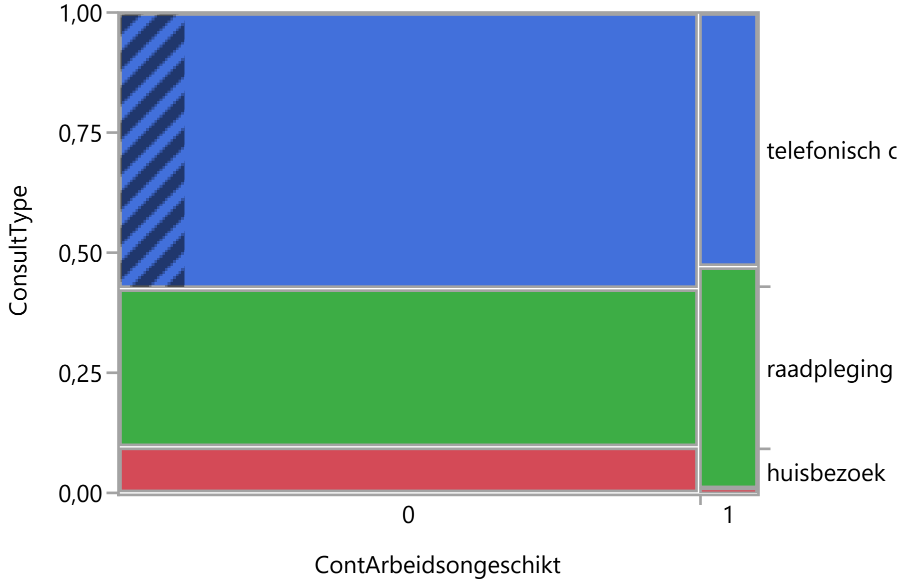

Contingency Table

ConsultType

ContArbeidsongeschikt	Count / Col %	huisbezoek	raadpleging	telefonisch consult	Total
0	Count	1403	4649	8165	14217
	Col %	98,80	87,54	91,49	
1	Count	17	662	759	1438
	Col %	1,20	12,46	8,51	
Total		1420	5311	8924	15655

Tests

N	DF	-LogLike	RSquare (U)
15655	2	117,29205	0,0083

Test	ChiSquare	Prob>ChiSq
Likelihood Ratio	234,584	<,0001*
Pearson	182,041	<,0001*

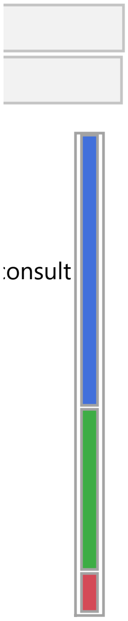
:onsult

Local Data Filter

15655 matching rows

☐ Inverse

Year (2)

2019 | 2020

Contingency Analysis of ConsultType By EDReferal

Mosaic Plot

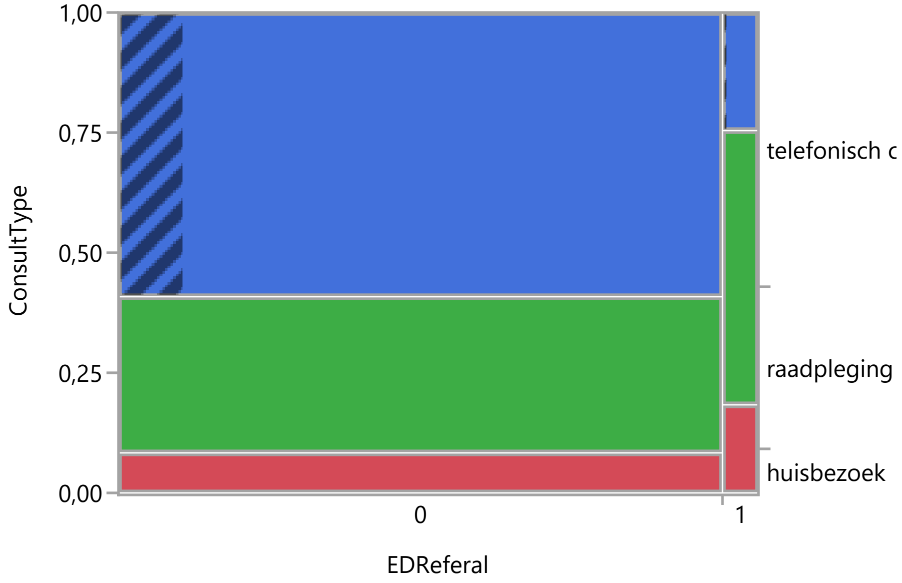

Contingency Table

EDReferal	Count / Col %	huisbezoek	raadpleging	telefonisch consult	Total
0	Count	1260	4815	8712	14787
	Col %	88,73	90,66	97,62	
1	Count	160	496	212	868
	Col %	11,27	9,34	2,38	
Total		1420	5311	8924	15655

Tests

N	DF	-LogLike	RSquare (U)
15655	2	203,69581	0,0144

Test	ChiSquare	Prob>ChiSq
Likelihood Ratio	407,392	<,0001*
Pearson	405,942	<,0001*

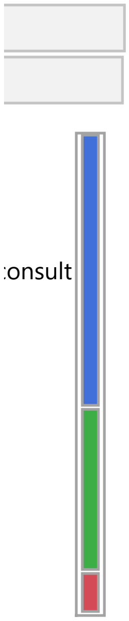
consult

Local Data Filter

15655 matching rows

☐ Inverse

Year (2)

2019 | 2020

Contingency Analysis of ConsultType By GPReferal

Mosaic Plot

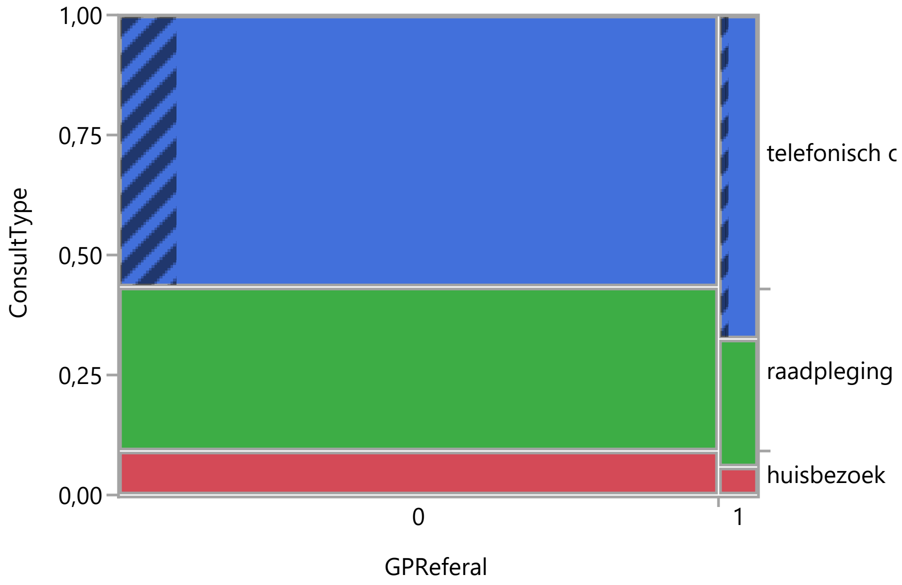

Contingency Table

ConsultType

GPReferal	Count<br>Col %	huisbezoek	raadplegin<br>g	telefonisch<br>consult	Total
	0	1363<br>95,99	5051<br>95,10	8272<br>92,69	14686
	1	57<br>4,01	260<br>4,90	652<br>7,31	969
	Total	1420	5311	8924	15655

Tests

N	DF	-LogLike	RSquare (U)
15655	2	23,866789	0,0017

Test	ChiSquare	Prob>ChiSq
Likelihood Ratio	47,734	<,0001*
Pearson	46,051	<,0001*

Local Data Filter

15655 matching rows

☐ Inverse

Year (2)

2019 | 2020

Contingency Analysis of ConsultType By WeekendNr

Mosaic Plot

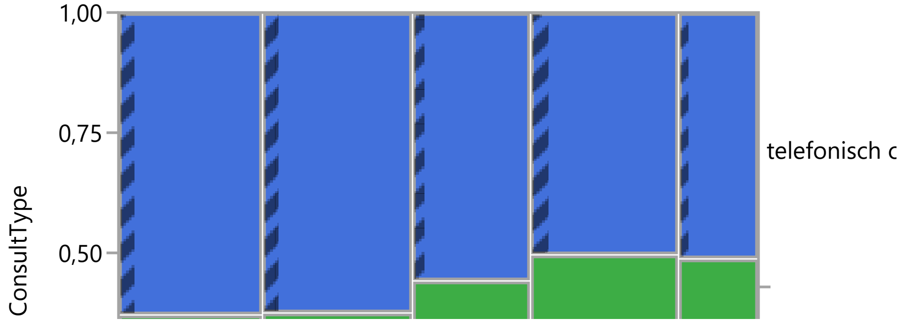

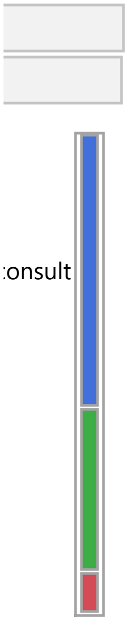

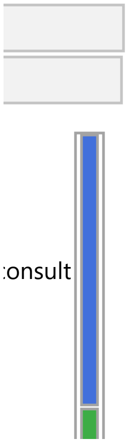

Contingency Analysis of ConsultType By WeekendNr

Mosaic Plot

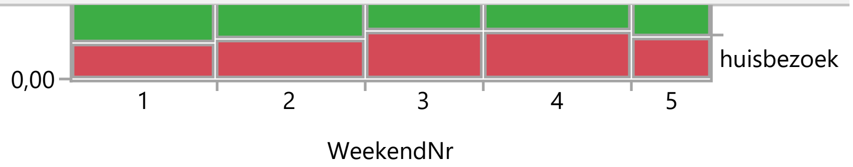

Contingency Table

ConsultType

WeekendNr	Count / Total % / Col % / Row %	huisbezoek	raadpleging	telefonisch consult	Total
1	Count	274	1051	2239	3564
	Total %	1,75	6,71	14,30	22,77
	Col %	19,30	19,79	25,09	
	Row %	7,69	29,49	62,82	
2	Count	309	1062	2259	3630
	Total %	1,97	6,78	14,43	23,19
	Col %	21,76	20,00	25,31	
	Row %	8,51	29,26	62,23	
3	Count	292	994	1617	2903
	Total %	1,87	6,35	10,33	18,54
	Col %	20,56	18,72	18,12	
	Row %	10,06	34,24	55,70	
4	Count	370	1424	1816	3610
	Total %	2,36	9,10	11,60	23,06
	Col %	26,06	26,81	20,35	
	Row %	10,25	39,45	50,30	
5	Count	175	780	993	1948
	Total %	1,12	4,98	6,34	12,44
	Col %	12,32	14,69	11,13	
	Row %	8,98	40,04	50,98	
Total	Count	1420	5311	8924	15655
	Total %	9,07	33,93	57,00	

Tests

N	DF	-LogLike	RSquare (U)
15655	8	97,781475	0,0069

Test	ChiSquare	Prob>ChiSq
Likelihood Ratio	195,563	<,0001*
Pearson	195,696	<,0001*

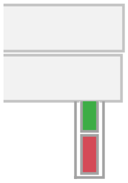

Local Data Filter

15655 matching rows

☐ Inverse

Year (2)

2019 2020

Contingency Analysis of PriorCall By SuspectedCovid 2

Mosaic Plot

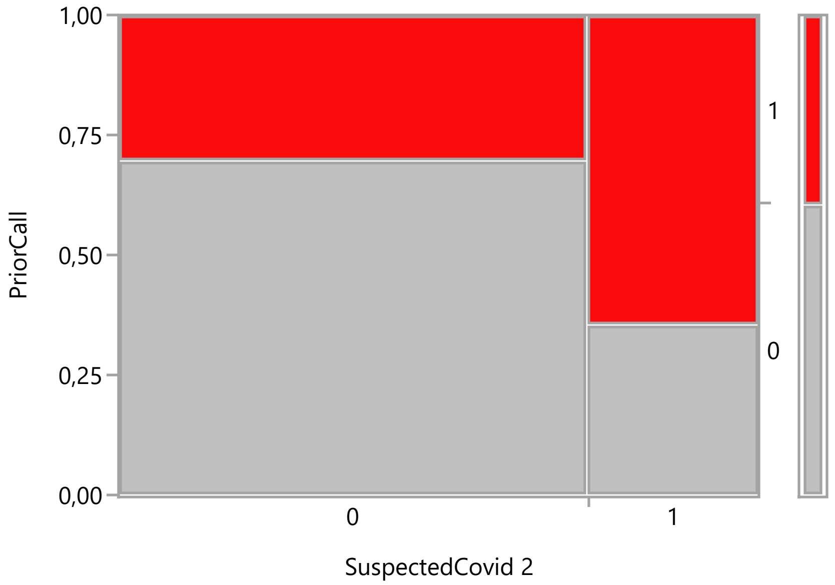

Contingency Table

PriorCall

SuspectedCovid 2	Count / Row %	0	1	Total
	0	3440	1493	4933
		69,73	30,27	
	1	642	1156	1798
		35,71	64,29	
	Total	4082	2649	6731

Tests

N	DF	-LogLike	RSquare (U)
6731	1	315,67548	0,0700

Test	ChiSquare	Prob>ChiSq
Likelihood Ratio	631,351	<,0001*
Pearson	639,294	<,0001*

Fisher's Exact Test	Prob	Alternative Hypothesis
Left	1,0000	Prob(PriorCall=1) is greater for SuspectedCovid 2=0 than 1
Right	<,0001*	Prob(PriorCall=1) is greater for SuspectedCovid 2=1 than 0
2-Tail	<,0001*	Prob(PriorCall=1) is different across SuspectedCovid 2

Local Data Filter

15655 matching rows

☐ Inverse

Year (2)

2019 | 2020

Contingency Analysis of PriorCall By SuspectedCovid 2

Mosaic Plot

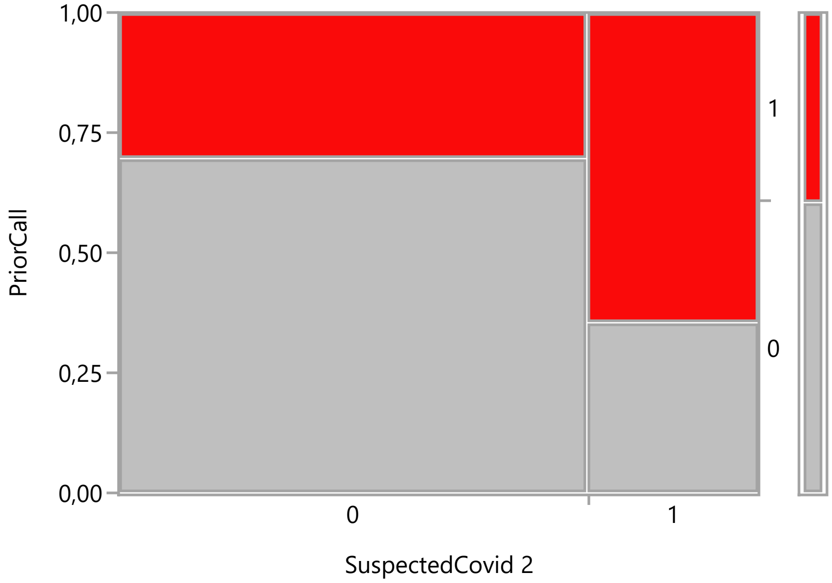

Contingency Table

PriorCall

SuspectedCovid 2	Count / Row %	0	1	Total
	0	3440	1493	4933
		69,73	30,27	
	1	642	1156	1798
		35,71	64,29	
	Total	4082	2649	6731

Tests

N	DF	-LogLike	RSquare (U)
6731	1	315,67548	0,0700

Test	ChiSquare	Prob>ChiSq
Likelihood Ratio	631,351	<,0001*
Pearson	639,294	<,0001*

Fisher's Exact Test	Prob	Alternative Hypothesis
Left	1,0000	Prob(PriorCall=1) is greater for SuspectedCovid 2=0 than 1
Right	<,0001*	Prob(PriorCall=1) is greater for SuspectedCovid 2=1 than 0
2-Tail	<,0001*	Prob(PriorCall=1) is different across SuspectedCovid 2

Local Data Filter

15655 matching rows

☐ Inverse

Year (2)

2019 2020

Contingency Analysis of ContArbeidsongeschikt By WeekendNr

Mosaic Plot

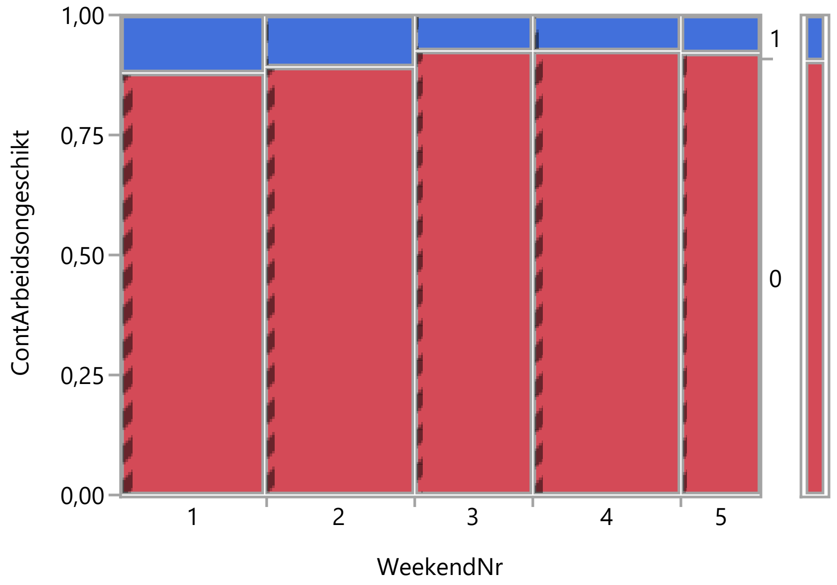

Contingency Table

ContArbeidsongeschikt

WeekendNr	Count / Row %	0	1	Total
	1	3142	422	3564
		88,16	11,84	
	2	3249	381	3630
		89,50	10,50	
	3	2688	215	2903
		92,59	7,41	
	4	3343	267	3610
		92,60	7,40	
	5	1795	153	1948
		92,15	7,85	
	Total	14217	1438	15655

Tests

N	DF	-LogLike	RSquare (U)
15655	4	32,914206	0,0069

Test	ChiSquare	Prob>ChiSq
Likelihood Ratio	65,828	<,0001*
Pearson	66,605	<,0001*

Local Data Filter

15655 matching rows

☐ Inverse

Year (2)

Oneway Analysis of Leeftijd By ConsultType

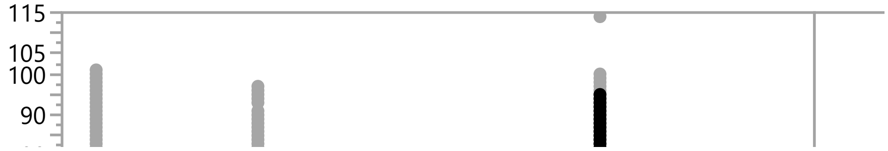

Local Data Filter

Oneway Analysis of Leeftijd By ConsultType

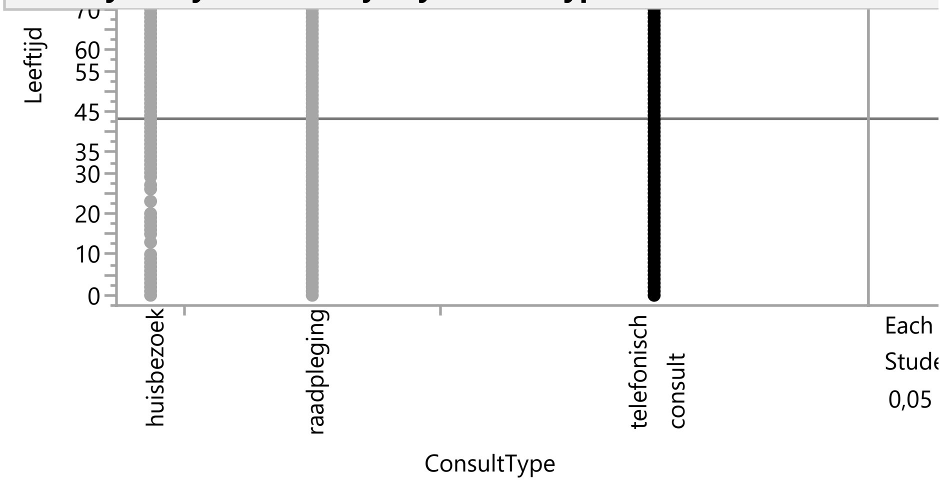

Means Comparisons

Comparisons for each pair using Student's t

Confidence Quantile

t	Alpha
1,96012	0,05

LSD Threshold Matrix

Abs(Dif)-LSD

	huisbezoek	telefonisch consult	raadpleging
huisbezoek	-1,682	31,440	33,714
telefonisch consult	31,440	-0,671	1,556
raadpleging	33,714	1,556	-0,870

Positive values show pairs of means that are significantly different.

Connecting Letters Report

Level				Mean
huisbezoek	A			73,758451
telefonisch consult		B		41,038100
raadpleging			C	38,705517

Levels not connected by same letter are significantly different.

Ordered Differences Report

Level	- Level	Difference	Std Err Dif	Lower CL	U
huisbezoek	raadpleging	35,05293	0,6830846	33,71401	[illegible]
huisbezoek	telefonisch consult	32,72035	0,6532619	31,43988	[illegible]
telefonisch consult	raadpleging	2,33258	0,3962578	1,55587	

Excluded Rows 8322

Pair
ent's t

pper CL	p-Value	
[illegible]6,39186	<,0001*	
[illegible]4,00082	<,0001*	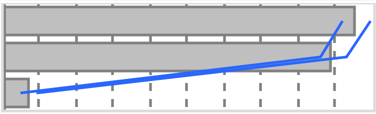
3,10929	<,0001*	

Local Data Filter

15655 matching rows

☐ Inverse

Year (2)

2019 2020

Contingency Analysis of ContArbeidsongeschikt By WeekendNr

Mosaic Plot

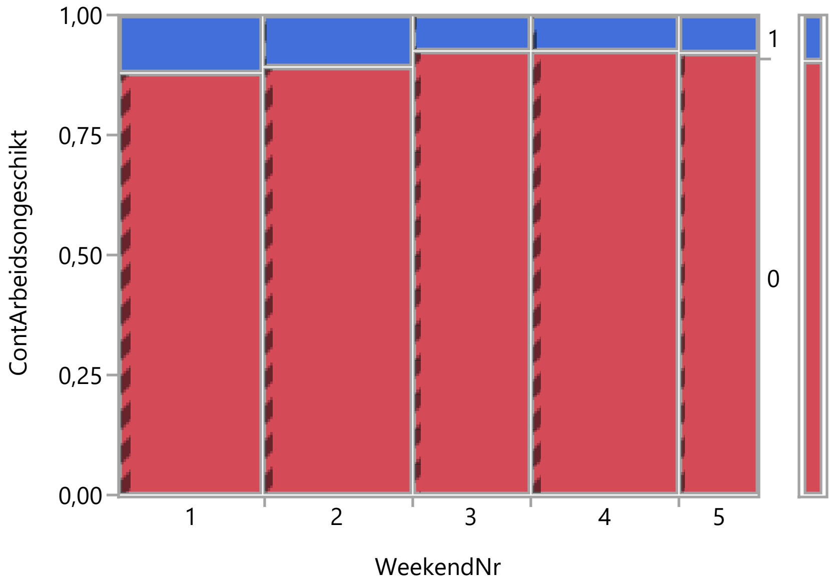

Contingency Table

ContArbeidsongeschikt

WeekendNr	Count<br>Row %	0	1	Total
	1	3142<br>88,16	422<br>11,84	3564
	2	3249<br>89,50	381<br>10,50	3630
	3	2688<br>92,59	215<br>7,41	2903
	4	3343<br>92,60	267<br>7,40	3610
	5	1795<br>92,15	153<br>7,85	1948
	Total	14217	1438	15655

Tests

N	DF	-LogLike	RSquare (U)
15655	4	32,914206	0,0069

Test	ChiSquare	Prob>ChiSq
Likelihood Ratio	65,828	<,0001*
Pearson	66,605	<,0001*

Local Data Filter

6692 matching rows

☐ Inverse

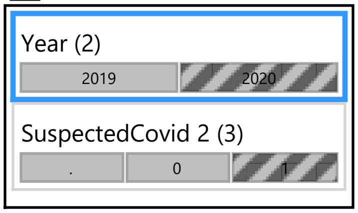

Contingency Analysis of EDReferal By ConsultType

Mosaic Plot

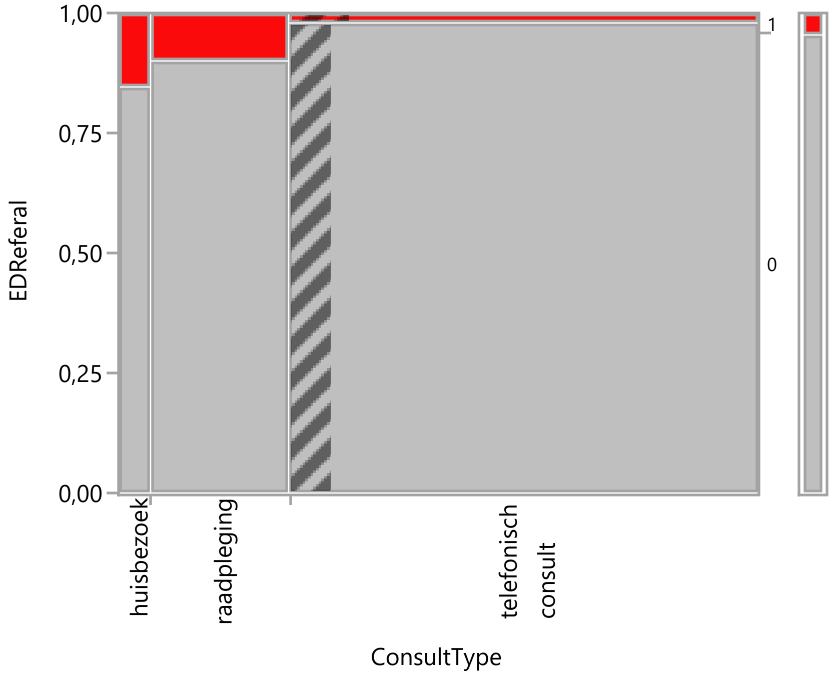

Contingency Table

ConsultType	Count / Row %	EDReferal 0	1	Total
	huisbezoek	287	51	338
		84,91	15,09	
	raadpleging	1319	141	1460
		90,34	9,66	
	telefonisch consult	4798	96	4894
		98,04	1,96	
	Total	6404	288	6692

Tests

N	DF	-LogLike	RSquare (U)
6692	2	108,27349	0,0912

Test	ChiSquare	Prob>ChiSq
Likelihood Ratio	216,547	<,0001*
Pearson	262,260	<,0001*
