# Supplementary material for: Organisation and characteristics of out-of-hours primary care during a COVID-19 outbreak: A real-time observational study
Source: PLoS One. 2020 Aug 13;15(8):e0237629. doi: 10.1371/journal.pone.0237629 (PMC7425859; doi:10.1371/journal.pone.0237629)
Supplement: Attachment — Submitted filename: Rebuttal letter.docx [file pone.0237629.s004.docx]

**Rebuttal letter**

Organisation and characteristics of out-of-hours primary care during a COVID-19 outbreak: a real-time observational study

Dear editor,

We thank the reviewers for their generous comments on the manuscript and have edited the manuscript accordingly. For your convenience we have made a table with the suggestions of the reviewers and our adaptations/comments.

| **Comment** | **Our adaptation and/or comment** |
| --- | --- |
| Change competing interests statement | We have added the suggestion to the cover letter. For clarity we have also copied our data availability statement to the cover letter. |
| Lacking in in-depth discussion about how this data might have larger consequences to changing health care system in Belgium during pandemic | This suggestion is similar to more specific critics of the second reviewer so we have commented those |
| Page 5 (Sec Methods): how are the other 77% of the Flemish population covered? It seems to be quite a large number of GPs per centre, are they all on duty in shifts? Do they otherwise work full time as GPs and cover the same population? | We have added the requested information. We have clarified that the studied GPCs cover 23% of the Flemish population. The remaining 77% is covered by GPCs we could not include or have the old system of rota groups for on call duties. We believe adding information about the large amount of included GPs would make the methodology section to long but provide it here for your interest: Belgium has got a rather large amount of GPs per capita and the included GPCs have a lot of part-time working GPs and trainees because of the proximity of two academic departments. GPs with a chronic illness or above 65 years of age are not obliged to work in the GPCs, all the others are. |
| Page 7 / Fig 1: the representation and explanation of the process during the pandemic should be clearer. All patients are advised to call first? | We have entirely rewritten this paragraph and simplified Fig 1. It is now more clear that all patients need to call (but some will only speak with a call taker). |
| It is stated on page 10, for example, that 30% did not have a telephone consultation. | We clarified this statement: 433 (30%) only spoke with a call taker |
| Please explain in more detail how you differentiate triage from consultation. | New sentence: “In two GPCs, the call takers refer selected patients directly to the ED (using a local triage protocol) while all other GPCs do not perform any kind of triage.” |
| How and when where patients informed about the new process? | Added to the introduction: The government’s recommendation to always call a doctor prior to going to a practice or ED was omnipresent in the media. |
| Do you have time stamps / durations for the call handling and different consultation types? Did the durations differ in the two years? Are telephone consultations significantly faster than physical consultations? | We do not have this data, added to limitations. |
| What was the staff utilisation in the two periods? | The GPCs definitely increased their staff (GPs and call takers/receptionists) but we do not have data proving this, neither was it part of our questionnaire. We have added this to the limitations. |
| Page 8 (Sec Characteristics, first paragraph): What were the remaining 3%? | There is not remaining 3%, we have clarified this sentence. |
| Page 10, Table 2: Why weren’t there any telephone consultations last year? Was it not possible to call the centre before the pandemic? Was everyone seen who called the centre? | Added to the introduction and to the footnote of this table: “Before the pandemic, remote consultations were neither reimbursed nor deontologically allowed.” |
| Page 10, Table 2: could you provide some numbers on the pandemic for the 5 weeks you studied to get a better idea about the situation in Belgium during that time? From the news we got the impression that Belgium was hit comparatively hard by the virus. | We have extended the epidemiological information in the introduction. Belgium surely was hit hard but we have chosen not to compare these data to other countries or regions because Belgium is one of the few countries including many suspected cases to the total number of cases. Currently there are no peer reviewed publications available about the comparison of the burden of this pandemic to individual countries or regions. It would be more correct to compare Belgium to New-York or Northern Italy then to compare the numbers to bigger countries. |
| Page 10, last sentence: do you have any explanation why ear-related diagnoses are on the list of those with the highest decline? From my experience, I would have imagined something like back pain or headaches etc. to be on that list. | There was a decline for back pain (included in the ICPC-chapter Musculoskeletal) and headache (chapter Neurological) but similar to the overall decline so we do not discuss them separately.  Added to the discussion: “The more pronounced decline of ophthalmological and digestive diagnoses is probably due to the reduced amount of other infections caused by the lock-down.” |
| From your findings, would you conclude that the centres should keep telephone consultations as an option even after COVID-19? Can you make conclusions based on your findings and your data? | Our data do not allow for definitive conclusions but are an interesting starting point. Added to discussion: “The successful implementation of telephone consultations opens new possibilities for the post COVID-19 era but should be studied more profoundly, especially regarding patient’s safety.” |
| Can you make any remarks on other European countries that also provide out-of-hours care by GPs like Germany? Did you find any papers or comments on that? Would you say that the care process should be transferable to other countries and would you suggest to use it? | We have entirely rewritten the first paragraph of the Discussion with additional references. This study alone does not allow for a recommendation on the transfer of this care process to other countries although is surely brings in one argument. |
| I am missing an outlook at the end of the paper. What should follow-up research study? How can the results be used in future research? | Such an outlook has been added to the final paragraph of the discussion. |

We hope that the manuscript is now suitable for publication in Plos One.

Sincerely,

The authors
