# Supplementary material for: Organisation and characteristics of out-of-hours primary care during a COVID-19 outbreak: A real-time observational study
Source: PLoS One. 2020 Aug 13;15(8):e0237629. doi: 10.1371/journal.pone.0237629 (PMC7425859; doi:10.1371/journal.pone.0237629)
Supplement: Attachment — Submitted filename: Second Rebuttal letter.docx [file pone.0237629.s005.docx]

**Rebuttal letter**

Organisation and characteristics of out-of-hours primary care during a COVID-19 outbreak: a real-time observational study

Dear editor,

We thank the reviewers for their additional comments on the manuscript and have edited the manuscript accordingly. We believe this paper is now ready for publication and will contribute to the wide diversity of knowledge about COVID-19 distributed by PLoS ONE as it is the first study within the COVID-19 collection with a primary care perspective. For your convenience we have made a table with the suggestions of the reviewers and our adaptations/comments.

| **Comment** | **Our adaptation and/or comment** |
| --- | --- |
| In order to provide a more complete information to our readers on the topic, we would like to emphasize the importance to cross referencing very recent material on the same topic published in "PLoS ONE ". | We have added a recent PLoS ONE study to the Methodology section (Grant MC, Geoghegan L, Arbyn M, et al. The prevalence of symptoms in 24,410 adults infected by the novel coronavirus (SARS-CoV-2; COVID-19): A systematic review and meta-analysis of 148 studies from 9 countries. PLoS One. 2020;15(6):e0234765. Published 2020 Jun 23. doi:10.1371/journal.pone.0234765) |
| Editor: add “Clinical features and short-term outcomes of 102 patients with corona virus disease 2019 in Wuhan, China. Clinical Infectious Diseases.” in the revision text | We have added this reference to the introduction |
| The information and references about other countries that were added to the discussion might be better moved to the intro section as part of the relevant literature. | We have moved this paragraph as requested |
| The last sentence of the added outlook should be revised. | This sentence was indeed incomplete and has been corrected |
| The paper needs final proofreading. | We have proofread the entire article and made some improvements here and there |

We hope that the manuscript is now suitable for publication in Plos One.

Sincerely,

The authors
